# PIGN spatiotemporally regulates the spindle assembly checkpoint proteins in leukemia transformation and progression

**DOI:** 10.1038/s41598-021-98218-y

**Published:** 2021-09-24

**Authors:** Emmanuel K. Teye, Shasha Lu, Fangyuan Chen, Wenrui Yang, Thomas Abraham, Douglas B. Stairs, Hong-Gang Wang, Gregory S. Yochum, Robert A. Brodsky, Jeffrey J. Pu

**Affiliations:** 1 Penn State Cancer Institute, Pennsylvania State University College of Medicine, Hershey, PA USA; 2 Renji Hospital, Shanghai Jiao Tong University School of Medicine, Shanghai, China; 3 Institute of Hematology, Peking Union Medical College, Tianjin, China; 4 Division of Hematology, Johns Hopkins Medicine, Baltimore, MD USA; 5 University of Arizona Cancer Center, 1515 N Campbell Avenue, #1968C, Tucson, AZ 85724 USA

**Keywords:** Cancer, Cell biology, Genetics, Molecular biology, Biomarkers, Diseases, Molecular medicine, Oncology, Pathogenesis, Risk factors

## Abstract

Phosphatidylinositol glycan anchor biosynthesis class N (PIGN) has been linked to the suppression of chromosomal instability. The spindle assembly checkpoint complex is responsible for proper chromosome segregation during mitosis to prevent chromosomal instability. In this study, the novel role of PIGN as a regulator of the spindle assembly checkpoint was unveiled in leukemic patient cells and cell lines. Transient downregulation or ablation of PIGN resulted in impaired mitotic checkpoint activation due to the dysregulated expression of spindle assembly checkpoint-related proteins including MAD1, MAD2, BUBR1, and MPS1. Moreover, ectopic overexpression of PIGN restored the expression of MAD2. PIGN regulated the spindle assembly checkpoint by forming a complex with the spindle assembly checkpoint proteins MAD1, MAD2, and the mitotic kinase MPS1. Thus, PIGN could play a vital role in the spindle assembly checkpoint to suppress chromosomal instability associated with leukemic transformation and progression.

## Introduction

The phosphoethanolamine (EtNP) transferase, phosphatidylinositol glycan anchor biosynthesis class N (PIGN) was initially identified as a glycosylphosphatidylinositol-anchor protein (GPI-AP) biosynthesis enzyme but has recently been shown in independent studies to prevent protein aggregation in the endoplasmic reticulum (ER) and suppress chromosomal instability (CIN)^1–3^. CIN commonly occurs in solid and hematological cancers and has been described as an enabling characteristic and a hallmark of cancer^4,5^. Moreover, increased frequency of GPI-AP deficiency has been linked to genomic instability and carcinogenesis^6,7^. Multiple studies have associated germline mutations in PIGN with intellectual disability and multiple developmental defects^8–10^. Normal embryonic development is dependent on proper chromosomal segregation during meiotic cell division^11^. Interestingly, a couple of studies have reported concurrent intellectual disability or developmental defects and hematological malignancies including MDS^12,13^. We have previously examined the pathophysiological impacts of PIGN expression aberrations on leukemia progression, due to the role that GPI-APs play in the maintenance of cellular structure and protection, signal transduction, and enzymatic biological processes^14,15^. We linked PIGN gene expression aberrations to partial intron retentions between exons 14 and 15 that resulted in frameshifts and premature termination codons in a subset of myelodysplastic/acute myeloid leukemia (MDS/AML) patients^15^. Moreover, these PIGN gene expression aberrations were associated with CIN and leukemic transformation. Thus, a better understanding of the mechanism(s) involved is critical for therapeutic targeting.

The spindle assembly checkpoint (SAC) ensures proper chromosomal segregation during mitosis to protect the genome from alterations in chromosome copy number and ultimately CIN^16^. Dynamic signaling interactions between mitotic kinases and SAC proteins are involved in sensing and stalling mitotic exit until proper attachment of mitotic spindles to the chromosomes and bi-orientation of the chromosomes on the spindles are achieved^17,18^. Quality control checks during metaphase to anaphase transition ensure proper kinetochore-microtubule (k-Mt) attachment and tension required to pull apart the sister chromatids to opposite spindle poles equally^19^. However, CIN-positive cells may have excessively stable k-Mt attachments compared to CIN-negative cells that ultimately result in mitotic errors^18^. In the case of improper k-Mt attachment, the SAC is activated via MAD1 that then recruits MAD2 and converts it from its inactive open form (o-MAD2) to its activated closed conformation (c-MAD2) where it can self-dimerize at the kinetochore^20^. Aurora kinase B phosphorylates and activates MPS1 which in turn, phosphorylates KNL1 that then serves as a scaffold for the recruitment of additional SAC proteins, including MAD1, MAD2, BUB3, BUB1, BUBR1, and CDC2^19,21,22^. At this point, the SAC effector also referred to as the mitotic checkpoint complex (MCC) composed of activated MAD2, BUBR1, and BUB3 sequesters CDC20 and prevents it from binding to and co-activating the ubiquitin ligase APC/C^23^. APC/C activation is required to polyubiquitinate and degrade Cyclin B and securin: Securin inhibits separase, which cleaves the cohesin proteins that hold sister chromatids together^23–26^.

Altered expression of SAC-related genes has been linked to aneuploidy in multiple human cancers including AML^27^. Moreover, SAC-related proteins and those involved in cell cycle control, including TP53, BUBR1, BUB1, MAD2, AURKA, AURKB, and PLK1 are frequently dysregulated in AML^28–30^. We have previously linked PIGN expression aberration with CIN in the leukemic transformation of MDS to AML and presented preliminary evidence of the involvement of PIGN and the SAC protein MAD1^15^. In this current study, we sought to understand the mechanism(s) involved in the previous implications of PIGN in the suppression of CIN and leukemic transformation. We did this by examining the impact of PIGN modulation on SAC signaling while exploring the potential function of PIGN as an interacting partner and regulator of SAC signaling.

## Materials and methods

Refer to the supplementary file for detailed information.

### Patient selection

All patients in this study were evaluated at the Pennsylvania State University College of Medicine between January 2013 and August 2017. Informed consent was obtained from each patient before obtaining peripheral blood and bone marrow aspirates under an approved IRB protocol (No. 40969). All experimental protocols were approved by the Pennsylvania State University College of Medicine IRB. All methods were carried out per IRB and institutional biomedical research committee relevant guidelines and regulations. Patients were diagnosed according to WHO guidelines^31^.

### Cell culture

Leukemia and lymphoblastoid cell lines (i.e. HL60, K562, Jurkat, KCL22, KG1, and KG1a) were cultured with RPMI or IMDM supplemented with 20% FBS. HEK293 and HEK293 PIGN CRISPR/Cas9 knockout cells were grown in DMEM supplemented with 10% FBS. Healthy volunteer CD34+ mononuclear cells were maintained in DMEM/F12 supplemented with 10% FBS, 50 μM 2-mercaptoethanol, Glutamax (Life Technologies), MEM non-essential amino acids (Life Technologies), and StemMACS HSC expansion cocktail (Miltenyi Biotec). Cell lines were authenticated by short tandem repeat (STR) profile analysis using Geneprint 10 System Kit (Promega) and compared to known ATCC fingerprints (ATCC.org).

### Gene expression analyses

RT-qPCR experiments were conducted as previously described^15^. Total RNA was isolated from the cells using the RNeasy Mini Kit (Qiagen) according to the manufacturer’s protocol. Total RNA was reverse transcribed using the High Capacity cDNA Reverse Transcription Kit (Life Technologies) on the Mastercycler Nexus (Eppendorf). The RT-qPCR step was conducted on the StepOnePlus Real-time PCR System (Applied Biosystems).

### HA-tag immunoprecipitation (IP)/ and co-immunoprecipitation (Co-IP) analyses

PIGN-HA IP/Co-IP experiments were conducted by transient transfection of CRISPR/Cas9 PIGN knockout HEK293 cells with the SRα promoter-driven expression vector pMEPuro3HAhPIGN or the empty vector without PIGN cDNA cloned^8^.

### Western blot analyses

Western blot analysis was conducted as previously described^15^. Briefly, total protein was extracted using RIPA cell lysis buffer supplemented with phosphatase inhibitor cocktail and protease inhibitor (Sigma). Whole-cell lysates were subjected to electrophoresis and transferred to an Immun-Blot PVDF Membrane (Bio-Rad). The membranes were blocked in 5% milk in TBS-T for 1 h at room temperature followed by overnight treatment at 4ºC with primary antibodies (1:500–1:1000) prepared in 5% milk/TBS-T. The membranes were then washed with TBS-T and incubated for 1–2 h with 1:5000–1:10,000 horseradish peroxidase-conjugated goat anti-rabbit (AP132P) or goat anti-mouse (AP124P) IgG secondary antibody (Millipore). Afterward, membranes were washed with TBS-T and treated with ECL Prime Western Blotting detection reagents (Amersham) to detect proteins.

### PIGN knockdown and CRISPR/Cas9 Knockout studies

RNAi-mediated PIGN knockdown experiments were conducted using the Nucleofector II Device (Amaxa) in conjunction with the Cell line Nucleofector Kit V reagent kit (Amaxa). CRISPR/Cas9 experiments were performed according to a modified LentiCRISPRv2 (plasmid #49535, Addgene) protocol^32^.

### Cell cycle analyses and SAC activation

**Figure 1:**
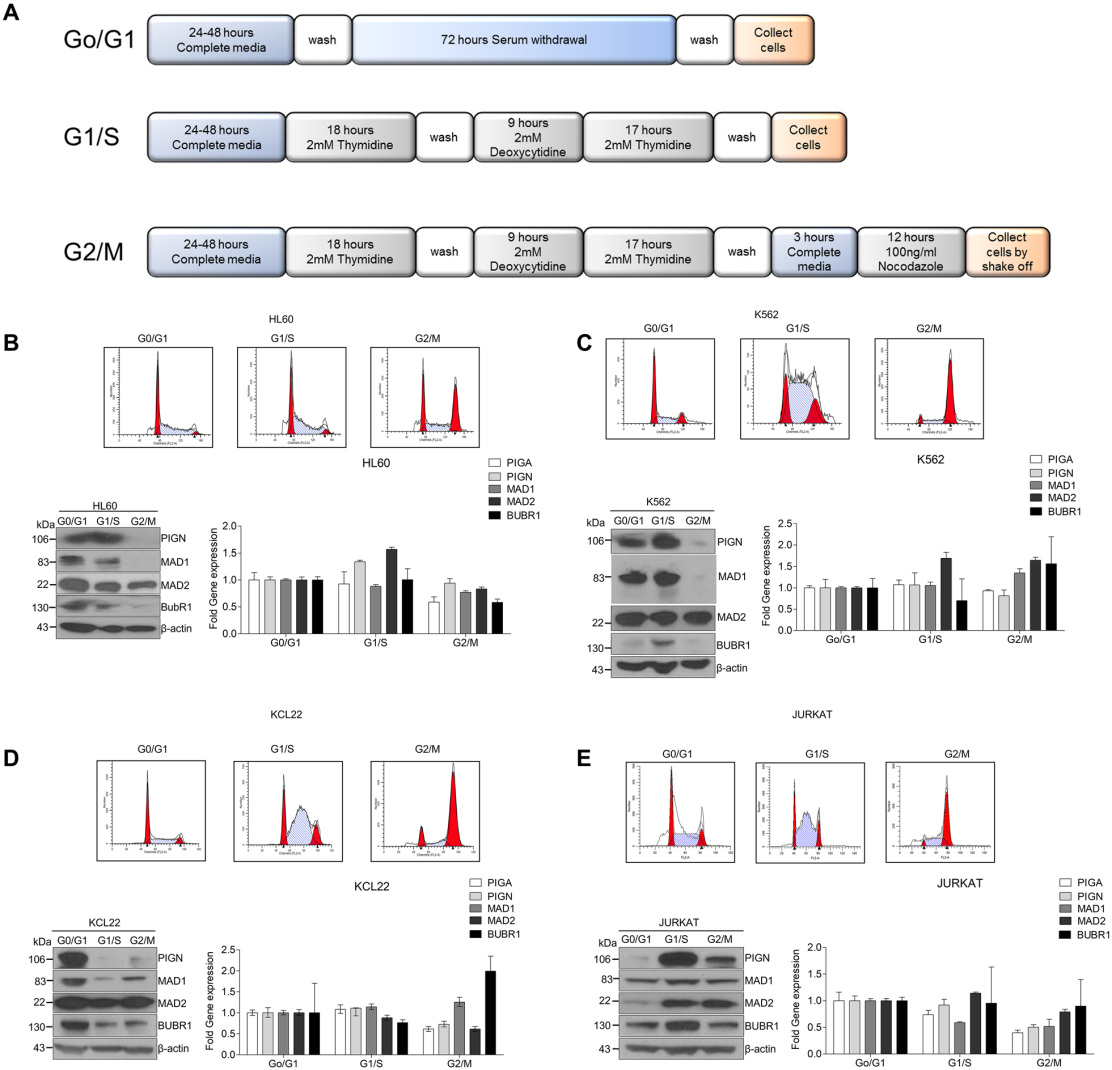
PIGN expression is cell cycle regulated. (**A**) Schematic of cell cycle synchronization protocols. Cell synchronizations were achieved using serum starvation for 72 h (Go/G1), double-thymidine block (G1/S), or double thymidine block and release followed by nocodazole treatment (G2/M). Cells were collected post-treatment for western blotting, RT-qPCR, and flow cytometry analyses. PIGN was expressed in a cell cycle-dependent manner in multiple cell lines: (**B**) HL60, (**C**) K562, (**D**) KCL22, and (**E**) JURKAT with suppressed expression from early S-phase to the G2/M phase. All western blot images were cut within the molecular weight ranges of the protein targets prior to hybridization. Images generated from these cut blots by scanning the developed films. Images were cropped and labeled using Adobe Photoshop CC 2017 (version 18). Where applicable, representative full images of the blots are presented in Figure 3 of the supplementary immunoblotting data.

Cell cycle synchronizations were performed as reported previously^33^. Serum starvation for 72 h was used to synchronize cells in G0 as described previously. Cells were synchronized at the Go/G1, S, and G2/M phases (Fig. 1A). Cell cycle synchrony was monitored using propidium iodide-stained cells with the BD FACSCalibur flow cytometer (BD Biosciences, San Jose, CA). All flow cytometry data were analyzed using ModFit LT V3.3.11 (Verity Software House, Topsham, Maine, USA). Protein lysates were obtained and used for Western blot analyses as described previously^15^.

### Immunofluorescence and confocal microscopy

For missegregation and colocalization analyses, cells were blocked in early S-phase via double-thymidine treatment and released for 6–8 h into the mitotic phase. Adherent cells were cultured on chambered slides. Cytospin was used to fix suspension cells onto slides. The slides were blocked with 2.5% normal goat serum diluted in PBS. The cells were treated at 4 °C overnight with primary antibodies followed by washing and treatment for 2 h at room temperature in the dark with secondary antibodies (1:1000). The slides were partially dried in the dark and mounted in Vectashield Hard Set mounting medium with DAPI (H-1500, Vector Laboratories). Images were acquired using the Leica SP8 inverted confocal microscope at the Microscopy Imaging. Three-dimensional image stacks were acquired in 0.15-μm steps using a × 40 1.4 N.A oil immersion objective. Deconvolution and analyses of image stacks were performed using the Huygens workstation (Scientific Volume Imaging B.V.), Imaris Microscopy Image Analysis (Bitplane AG), and Volocity 6.3 (PerkinElmer).

### Mitotic index and cell cycle frequency analyses

HEK293 cells were detached from the plate, washed with PBS, and passed through a cell filter to obtain single cells. Then, the cells were incubated with anti-phospho-Histone H3 (Ser10) (05–806, Millipore Sigma). Afterward, the cells were resuspended in 100 μL of PBS containing 1% BSA and Alexa 488-conjugated goat anti-mouse immunoglobulin G antibody (A32723, ThermoFisher Scientific) (1:300) and incubated at room temperature in the dark for at least 30 min. After incubation, the cells were resuspended in PI/ RNase A and incubated at room temperature in the dark for at least 30 min. Finally, the cells were analyzed via flow cytometry analyses on a 2-color AlexaFluor488 (FITC) vs PI or stored at 4 °C until FACS analysis.

### Statistical analyses

GraphPad Prism 8 and Microsoft Excel were used for statistical analyses. Two-tailed Student’s t-tests, one-way or two-way ANOVA followed by Tukey’s post hoc tests for multiple comparisons; p-values ≤ 0.05 were considered statistically significant.

## Results

### PIGN expression is cell cycle-regulated

To clarify the mechanistic role of PIGN in the maintenance of chromosomal stability, we examined the impact of PIGN on cell cycle signaling. The SAC is responsible for proper chromosomal segregation^17^. We performed cell cycle synchronization experiments using myeloid and lymphoblastoid cell lines and examined PIGN expression at different stages of the cell cycle. Cell cycle synchronization experiments were conducted by blocking cell cycle progression at G0/G1, G1/S, and G2/M phases in K562, HL60, KCL22, and Jurkat cells via serum starvation, double-thymidine, and nocodazole treatment respectively (Fig. 1A). The HL60 cells, despite multiple experiments, did not respond as well to cell synchronization as the other cell lines examined. Nonetheless, synchronization of these myeloid and lymphoblastoid cell lines at various cell cycle stages revealed that PIGN was differentially expressed during the cell cycle (Fig. 1B–E). Overall, PIGN and MAD1 were least expressed during the G2/M phase of the cell cycle, and protein levels of PIGN steadily decreased from early S-phase into the G2/M phase in almost all the cell lines that were examined. Also, the pattern and level of PIGN expression were comparable to the expression of other components of the SAC particularly, MAD1 and BUBR1. Thus, the expression of PIGN is cell cycle-regulated, similar to the SAC proteins under consideration.

### PIGN loss or suppression results in the dysregulation of SAC components

**Figure 2:**
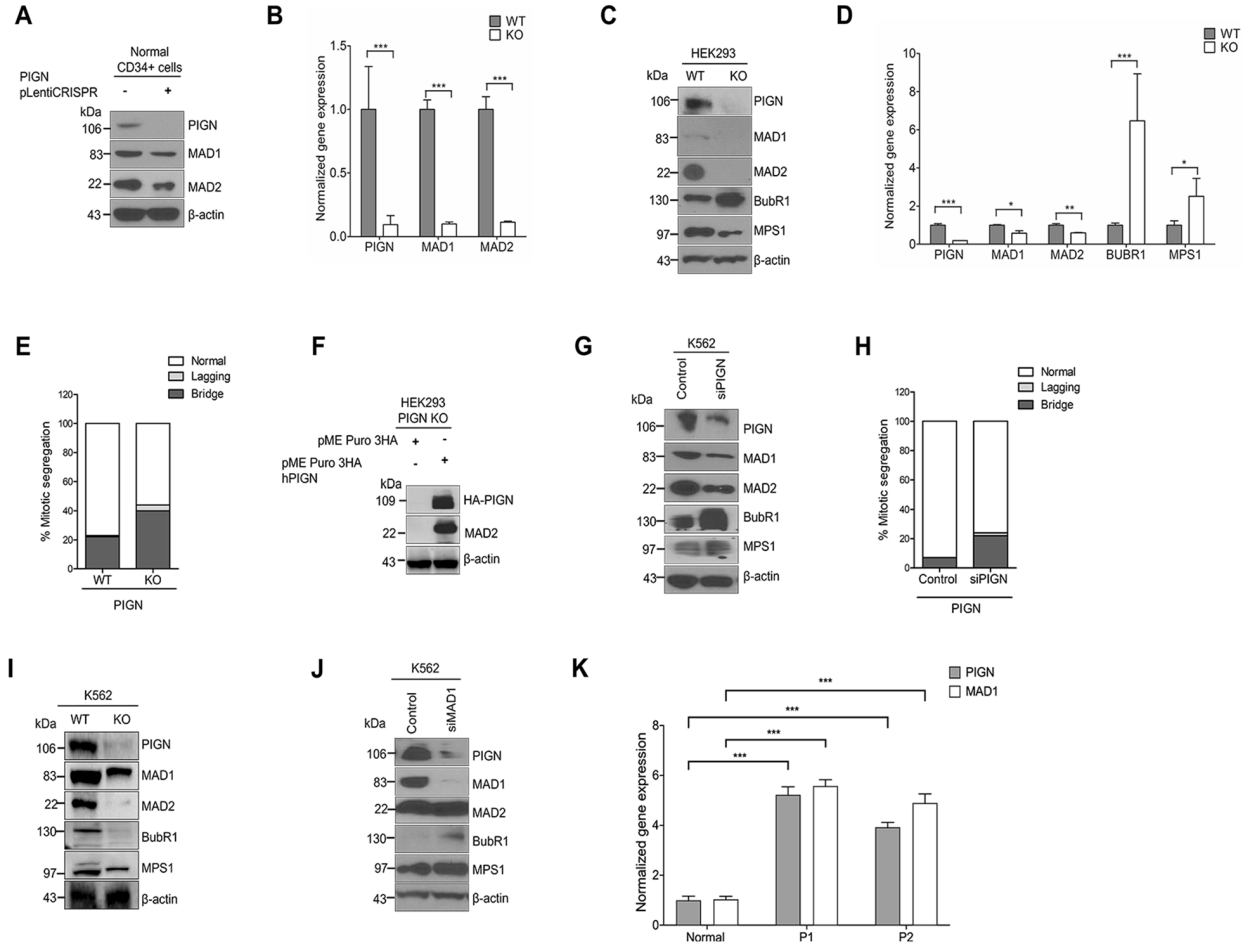
PIGN loss or suppression results in the differentially disrupted expression of SAC components. (**A**) CRISPR/Cas9 ablation of PIGN led to MAD1 and MAD2 downregulation in CD34+ mononuclear cells from a healthy donor. (**B**) Gene and protein expressions of MAD1 and MAD2 were significantly (***p < 0.0001) impacted by PIGN loss in CD34+ mononuclear cells from a healthy donor. (**C**,**D**). Complete loss of PIGN via CRISPR/Cas9 ablation (KO) was associated with downregulation of MAD1, MAD2, and MPS1 while causing an upregulated gene (***p < 0.0001) and protein expression of BUBR1 in HEK293 cells. PIGN loss resulted in significant repression (*p < 0.05) of MAD1 and MAD2 gene expression but led to MPS1 gene upregulation (*p < 0.05). (**E**) PIGN loss (KO) increased the frequency of segregation errors in HEK293 cells. (**F**) Ectopic overexpression of PIGN restored MAD2 expression in HEK293 PIGN KO cells. (**G**) RNAi-mediated PIGN suppression resulted in MAD1 and MAD2 downregulation but increased expression of BUBR1 and MPS1 expression in K562 cells. (**H**) MAD1 suppression was accompanied by a corresponding decrease in PIGN protein expression in K562 cells. (**I**) CRISPR/Cas9 ablation of PIGN (K562 KO) resulted in MAD1, BUBR1, and MPS1 downregulation in K562 cells. (**J**) RNAi-mediated MAD1 suppression in K562 cells resulted in MAD1 downregulation while causing an upregulation in BUbR1 and MPS1 expression. (**K**) AML-MRC patient CD34+ PBMCs (P1 and P2) with PIGN partial intron retentions showed a significant (***p < 0.0001) increase in PIGN and MAD1 gene expressions compared to non-leukemic control cells from a healthy donor. All western blot images were cut within the molecular weight ranges of the protein targets prior to hybridization. Images generated from these cut blots by scanning the developed films. Images were cropped and labeled using Adobe Photoshop CC 2017 (version 18). Where applicable, representative full images of the blots are presented in Figure 2 of the supplementary immunoblotting data.

RNAi-mediated knockdown and CRISPR/Cas9 ablation of PIGN were employed to assess the impact of PIGN loss or downregulation on the SAC. CRISPR/Cas9-mediated loss of PIGN resulted in the suppression of MAD1 and MAD2 in CD34+ mononuclear cells derived from the peripheral blood sample of a healthy individual (Fig. 2A,B). A similar observation was made in HEK293 CRISPR/Cas9 PIGN knockout cells (KO): PIGN loss in the HEK293 cells resulted in the suppression of MAD1, MAD2 expression but an upregulation in BUBR1 expression (Fig. 2C,D). PIGN loss was accompanied by an increased frequency of CIN-associated segregation errors in the HEK293 KO cells (Fig. 2E). However, ectopic overexpression of PIGN restored MAD2 expression, which seemed to be lost in the PIGN KO cells (Fig. 2C,F).

RNAi-mediated suppression of PIGN in K562 myeloid leukemia cells caused MAD1 and MAD2 suppression but BUBR1 upregulation that was accompanied by an increase in the frequency of segregation errors (Fig. 2G,H). Moreover, MAD1, MAD2, and MPS1 were downregulated with PIGN loss in K562 myeloid leukemia cells (Fig. 2I). To further elucidate the regulatory relationship between PIGN and the SAC, RNAi-mediated knockdown of MAD1 was employed. This resulted in PIGN downregulation accompanied by BUBR1, and MPS1 upregulation but no detectable change was observed in MAD2 expression (Fig. 2J). The suppression of PIGN expression with MAD1 knockdown unveiled a unique reciprocal regulatory relationship between MAD1 and PIGN. Also, PIGN gene expression upregulation was associated with an increase in MAD1 gene expression in AML with myelodysplasia-related changes (AML-MRC) patients (P1 and P2) bearing a PIGN mutation (i.e. partial intron retention mutation between exons 14 and 15) (Fig. 2K)^15^. Overall, a regulatory link seems to exist between PIGN and the SAC.

### PIGN spatiotemporally interacts with SAC components

**Figure 3:**
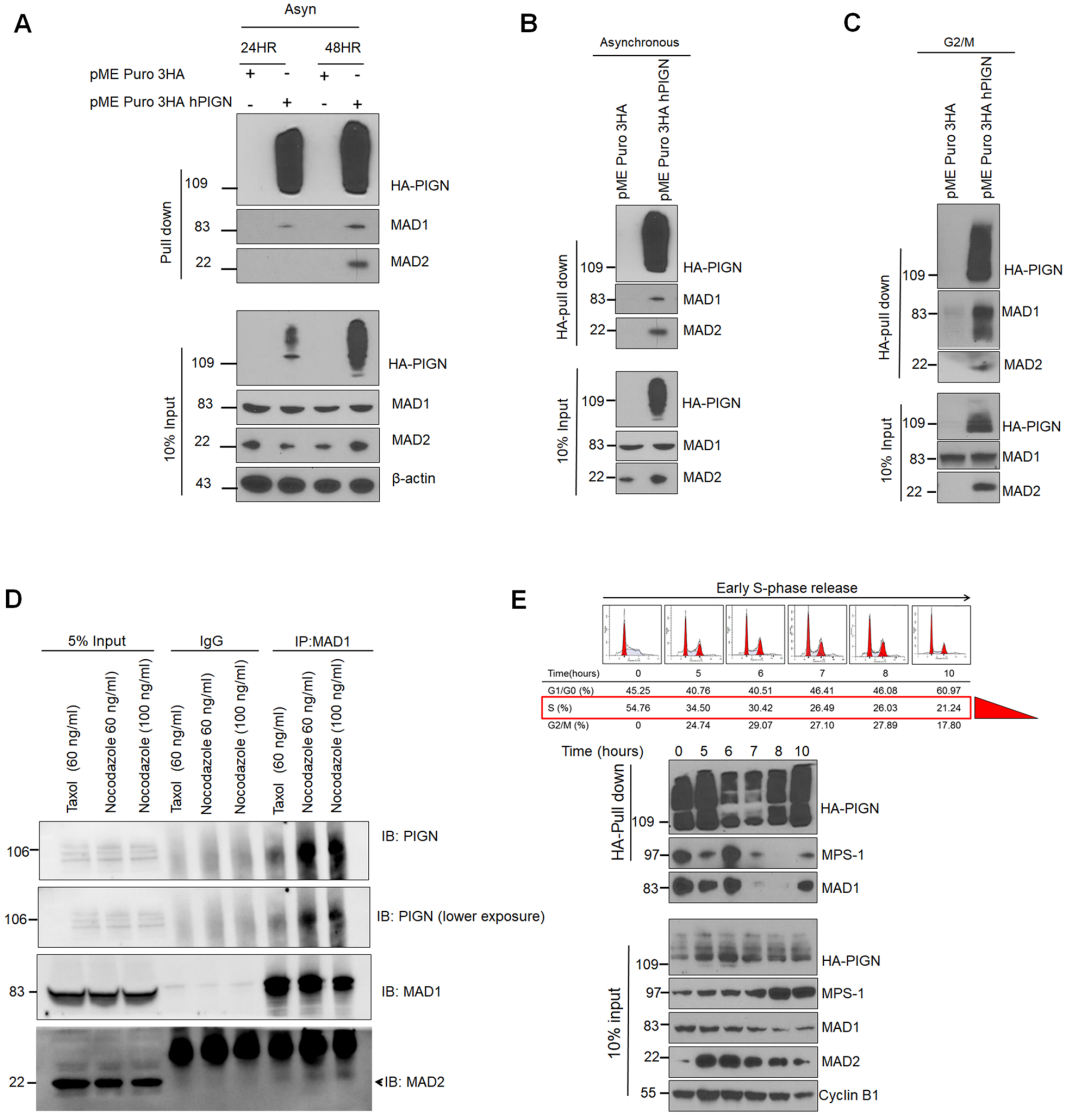
PIGN forms a complex with SAC components. MAD1 and MAD2 were co-purified with PIGN in an HA-tag pull-down assay in PIGN null HEK293 cells ectopically expressing 3HA-tagged PIGN. (**A,B**) HA-tag IP in asynchronous cells indicated optimal 3HA-tagged PIGN expression and co-purification with MAD1 and MAD2 at the 48-h time point. (**C**) MAD1 and MAD2 were co-purified with PIGN in G2/M synchronized cells. (**D**) Co-immunoprecipitation (Co-IP) experiment showed that PIGN endogenously interacted with MAD1 and MAD2 during SAC activation via Taxol (60 ng/µl) or nocodazole (60–100 ng/µl) treatment of K562 cells for 12 h. (**E**) HA-tag pull-down assay with cells released from early S-phase into mitosis indicated PIGN co-purification with MAD1 and MPS1. Inputs represent 5–10% of total protein lysate. All western blot images were cut within the molecular weight ranges of the protein targets prior to hybridization. Images generated from these cut blots by scanning the developed films. Images were cropped and labeled using Adobe Photoshop CC 2017 (version 18). Where applicable, representative full images of the blots are presented in Figure 3 of the supplementary immunoblotting data.

HA-tag IP experiments were conducted using CRISPR/Cas9 PIGN KO HEK293 cells transfected with an N-terminus HA-tagged PIGN plasmid. The results of these experiments revealed a complex involving PIGN, MAD1, and MAD2 by 48 h post-transfection.

(Fig. 3A,B). Thus, PIGN may form a complex with MAD1 and MAD2 during SAC activation. Since the SAC is activated during mitotic cell division, we verified whether this PIGN-inclusive complex was formed during mitosis. The results indicated that PIGN formed a complex involving MAD1 and MAD2 during the mitotic phase (Fig. 3C). This observation revealed PIGN as a novel interacting partner in G2/M synchronized cells. Co-IP studies were initially used to verify that the interaction between PIGN, MAD1, and MAD2 occurred endogenously during SAC activation. MAD1 co-precipitated with PIGN and MAD2 during drug-induced SAC activation (Fig. 3D). The two drugs (i.e. Taxol and Nocodazole) were used to target β-tubulin and halt mitotic division. However, there was an observable difference in the level of PIGN, MAD1, and MAD2 interactions between the Taxol-treated and the Nocodazole-treated cells. Nocodazole disrupts mitotic spindle function by reversibly interfering with microtubule dynamics and polymerization to induce mitotic arrest and as such induces a stronger SAC response, unlike Taxol which promotes the formation of highly stable microtubules that are resistant to depolymerization^34^.

Next, we sought to determine, whether these interactions were cell cycle-dependent and not limited to the mitotic stage of the cell cycle. To do this possibility, we synchronized cells in early S-phase (G1/S-phase) via double-thymidine block and release over a 10-h time course. We identified MAD1 and MPS1 as interacting partners of PIGN during this 10-h time course (Fig. 3E). Overall, PIGN may maintain chromosomal stability by forming a complex with the SAC proteins MAD1, MAD2, and MPS1 at various points during the cell cycle.

### PIGN colocalizes with SAC components during SAC activation and impacts mitotic exit

**Figure 4:**
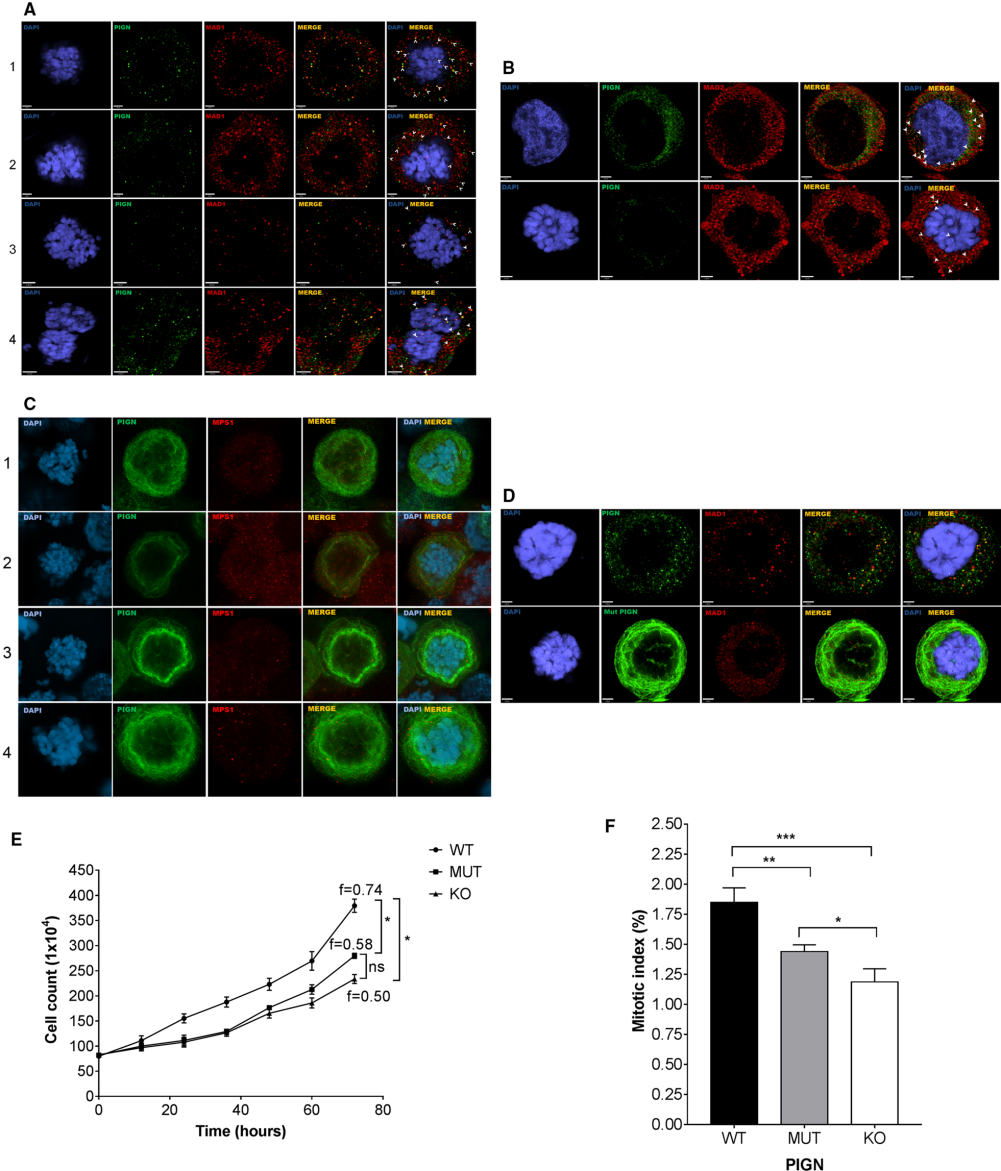
PIGN colocalizes with SAC components during SAC activation. Colocalization (yellow) of PIGN (green) with (**A**) MAD1 (42% ± 10%), (**B**) MAD2 (65% ± 9%) and (**C**) MPS1 (red) in HEK293 cells. HEK293 PIGN KO cells were transfected with pMEPuro3HAPIGN plasmid for 48 h followed by treatment with nocodazole (100 ng/µl) for 12 h. % Colocalization (mean % ± SEM) was calculated based on proportions of overlapping red and green voxels or the object Pearson correlation. (**D**) Colocalization of the exon 14/15 intron-retaining mutant PIGN (mut PIGN) with MAD1. The mutant plasmid was cloned by inserting a 38 bp partial intron sequence into the wild-type gene in the pMEPuro3HAPIGN plasmid via restriction enzyme digestion and re-ligation. The cells were incubated for 48 h followed by treatment with nocodazole (100 ng/μl) for 12 h. Cells transfected with either mutant or wild-type plasmid were fixed with 4% paraformaldehyde and treated with mouse anti-MAD, anti-MAD2 or anti-MPS1, and rabbit anti-HA, followed by treatment with fluorescently-labeled secondary antibodies. Chromosomes were stained with DAPI (blue). Laser scanning confocal microscopy was used to visualize the stained cells. Scale bars, 2–3 µm. The cells were fixed with 4% paraformaldehyde and treated with mouse anti-MAD, anti-MAD2 or anti-MPS1, and rabbit anti-HA, followed by treatment with the respective fluorescently-labeled secondary antibodies. Chromosomes were stained with DAPI (blue). Laser scanning confocal microscopy was used to visualize the stained cells, and image analyses were conducted using the Volocity 6.3 High-performance 3D imaging software (PerkinElmer). All images were deconvolved and imaging analyses performed with Huygens Professional version 19.04 (Scientific Volume Imaging, The Netherlands, http://svi.nl). Scale bars, 2-3 µm. N.D = not determined. (**E**) PIGN loss in HEK293 cells decreased cell cycle frequency in HEK293 cells. Cell cycle frequency (1/day) was significantly lower in HEK293 KO cells ectopically overexpressing the PIGN mutant (MUT) (*p = 0.0276) or empty vector (KO) control (*p = 0.0444) compared to those expressing wild-type PIGN (WT). Mean cell counts were obtained over 3 days at 12-h intervals in three separate experiments (n = 3). The cell cycle frequency (f) was calculated using the formula ${\mathrm{f}} = \left[ {\frac{{\ln {\mathrm{Nt}}/{\mathrm{No}}}}{\ln 2}} \right] \times \frac{1}{{\mathrm{t}}}$ derived from the formula N_t_ = N_0_ 2^tf^ where N_t_ is the number of cells at time t, N_0_ is the initial number of cells and f is the frequency of cell cycles per unit time. M. Beals, L. Gross, S. Harrell. 1999. Quantifying cell division. Error bars indicate mean and standard deviation. (**F**) Mitotic index was significantly reduced in HEK293 KO cells ectopically overexpressing mutant PIGN (MUT) (**p = 0.0056) or empty control vector (KO) (***p = 0.0004) compared to wildtype (WT) PIGN. Error bars are representative of the mean and standard error from the mean in three independent experiments (n = 3).

We used confocal microscopy to visualize the interaction between PIGN and SAC components, MAD1 and MAD2. Co-immunofluorescence analyses revealed a dynamic colocalization of PIGN and the aforementioned SAC proteins during mitosis specifically during prometaphase (Fig. 4A,B). Thus, the interactions between PIGN, MAD1, and MAD2 may be early events during SAC activation. PIGN and MAD1 showed cellular localization patterns commensurate with what has previously been reported^35^. We have previously linked a PIGN mutation (i.e. partial intron retention between exons 14 and 15) to leukemic transformation in AML-MRC patients^15^. Thus, we examined the impact of this mutation on the colocalization of PIGN with MAD1. Our results indicated that both the wild-type PIGN and the overexpressed mutant colocalized with MAD1 (Fig. 4C). Moreover, the presence of the intron-retaining mutant form of the PIGN protein (MUT) or PIGN ablation (KO) resulted in decreased mitotic indices compared to the wild-type PIGN protein (Fig. 4E,F).

## Discussion

**Figure 5:**
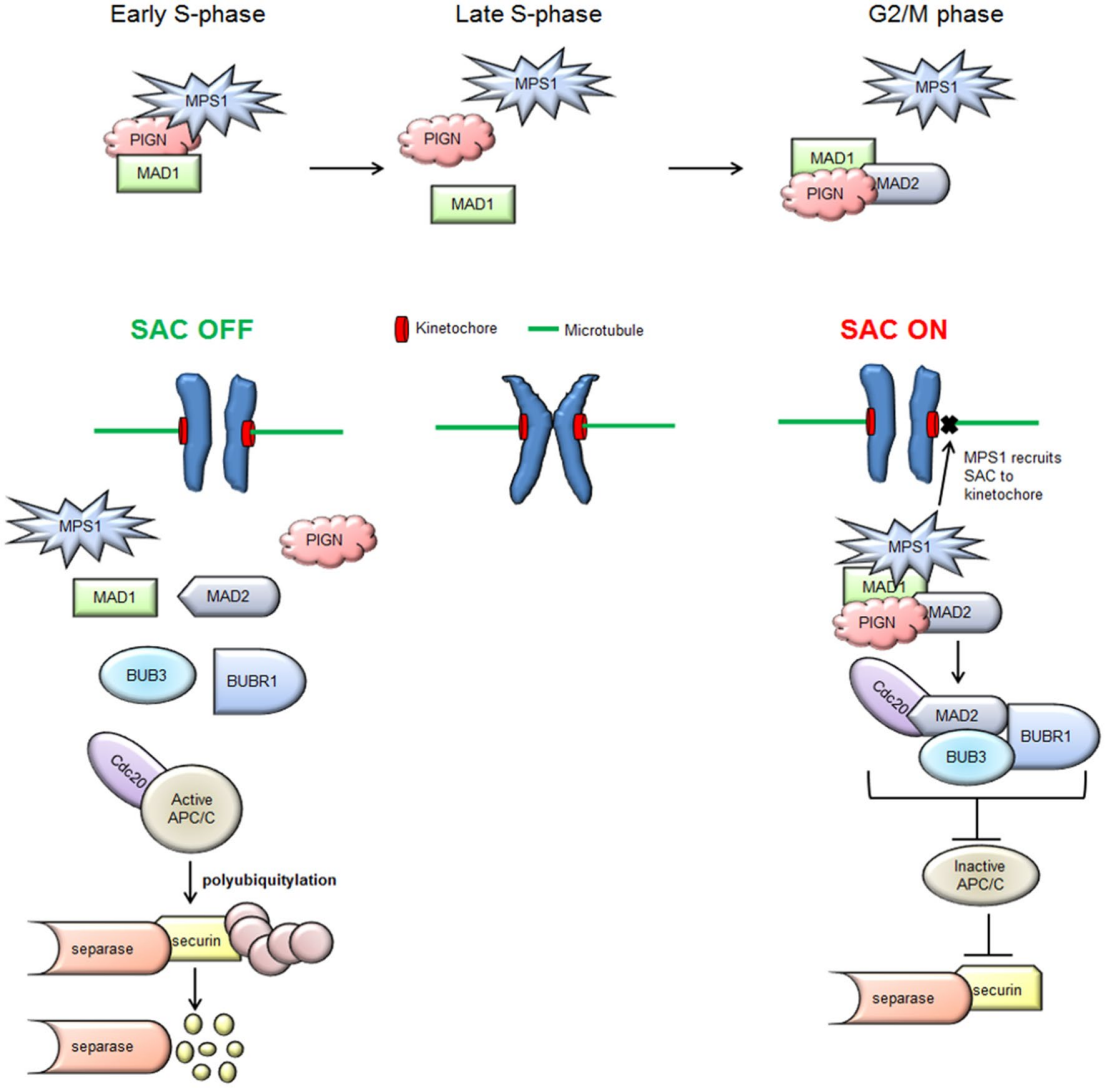
Simplified model of the spatiotemporal interaction between PIGN and SAC components during the cell cycle and SAC activation. PIGN interacts with SAC components in a time-dependent manner and may at some time points exclude interaction with MAD2.

Since its discovery as a CIN suppressor, the mechanism by which the loss of the GPI-AP biosynthesis protein, PIGN leads to CIN has remained elusive. Moreover, conflicting opinions exist in the literature with regards to the origins of CIN, whether pre-mitotic or mitotic^3,18,36^. In our prior study, PIGN loss triggered genomic instability and was linked to leukemic transformation in AML-MRC patients and an MDS transformation cell line model^15^. CIN has been implicated in leukemia progression and has been proposed as a therapeutic target^28,37–40^. In this study, we investigated the mechanistic basis of PIGN in CIN suppression and consequently, leukemic transformation. The cell cycle-dependent expression of PIGN in the context of SAC protein expression and relatively higher expressions of SAC proteins during the S-phase (Fig. 1) as well as interactions with PIGN from early S-phase through mitosis (Fig. 3E) confirmed previous studies that have demonstrated that the SAC can be activated before to mitosis and as early as during the S-phase^41,42^. These results offered evidence of spatiotemporal interactions between PIGN and SAC components and may reveal the order in which these interactions occur (Fig. 5).

PIGN and MAD1 are necessary for proper chromosomal alignment and segregation^3,43^. The suppression or loss of PIGN resulted in MAD1 downregulation and vice versa, accompanied by an increased frequency of segregation errors (Fig. 2A–J). We have previously reported increased PIGN gene expression in AML-MRC patient CD34+ PBMCs with partial intron retentions in the PIGN gene compared to normal non-leukemic control cells^15^. In this current study, a significant increase in PIGN expression was associated with a significant increase in MAD1 expression in AML-MRC patients known to possess partial intron retentions in the PIGN gene. Additionally, we observed complex formation and/or colocalization involving PIGN and the SAC proteins, MAD1, MAD2, and MPS1. Thus, it is important to investigate the relevant protein sequences involved in these interactions and to establish how PIGN depletion affects the recruitment of SAC proteins to the kinetochore. This is vital because of the dynamic localization of MAD1 between the spindle attached-kinetochore and the spindle poles for the activation and silencing of the SAC respectively^17,20,44^. Nevertheless, our findings reveal the complementary role that PIGN and MAD1 could play in the co-stabilization of the SAC and regulating the availability and migration of SAC proteins to disrupted k-Mt. The interaction of PIGN with MAD1, reciprocal regulation between them as well as their similar pattern of cell cycle-regulated expression (Fig. 1,2) may indicate a direct effect related to the stability of SAC-related proteins, with MAD1 being the limiting factor in SAC activation^20,43,45^.

A degree of regulatory control seems to exist between PIGN and other SAC proteins including MAD2, BUBR1, and MPS1. PIGN ablation resulted in the suppression (Fig. 2A) or loss (Fig. 2C,F,I) of MAD2 which was rescued by ectopic overexpression of PIGN. PIGN ablation impacted BUBR1 expression, leading to an upregulation in BUBR1 expression in HEK293 PIGN KO cells similar to RNAi-mediated suppression of PIGN expression K562 cells (Fig. 2C,G). Conversely, CRISPR/Cas9-mediated PIGN ablation resulted in BUBR1 downregulation in the K562 myeloid leukemia cells (Fig. 2I). These variable observations could be cell context-related and/or attributable to PIGN availability (i.e. stable loss vs. transient suppression) in the cells under consideration. Moreover, other factors may be involved in the regulatory link between PIGN and BUBR1. Regardless, the downregulation of PIGN expression modulated BUBR1 expression. Aside from their role in the SAC, MAD2, and BUBR1 have been implicated in the timing of mitotic regulation^46^. Thus, PIGN regulation of MAD1 and BUBR1 expression may be indicative of regulatory control on mitotic timing. Moreover, PIGN loss modulated MPS1 protein expression. MPS1 gene expression seemed to be slightly upregulated despite a reduction in protein expression following PIGN loss in HEK293 cells (Fig. 2C,D). This disparity between gene and protein expression may be linked to the post-translational regulation of MPS1^47–49^. Also, PIGN KO cells and cells ectopically overexpressing the intron-retaining mutant form of PIGN (MUT), presented with a significantly lower cell cycle frequency and mitotic index compared to wild-type PIGN (WT)-expressing cells (Fig. 4E,F). A weakened SAC may result in mitotic slippage or cell death in acute leukemia^50^. Thus, it will be worth investigating in future studies whether the relatively lower mitotic index in the PIGN knockout cells is attributable to cell death.

Optimal expression, timely activation, modulation, and silencing of the SAC-related factors are very critical for the efficiency and the stepwise time-bound precision of cell cycle progression^17^. Thus, we sought to identify at which stage of the cell cycle these interactions occurred and other SAC components that may be involved. Despite its widespread cytoplasmic and ER localization, it is yet to be determined how PIGN may regulate the recruitment of SAC proteins to the kinetochore. Our observation of PIGN interaction with MAD1 and MPS1 from early S-phase revealed that a complex involving PIGN, MAD1, and MPS1 may be involved (Fig. 3E). Moreover, interactions between PIGN, MAD1, and MAD2 were observed in G2/M synchronized cells (Fig. 3C,D). Thus, MAD2 recruitment may be a later event following MAD1 and MPS1 recruitment. Overall, PIGN may interact with MPS1 and MAD1 in a ‘pre-SAC’ complex during early S-phase till late S-phase and form another complex involving MAD1 and MAD2 during mitosis (Fig. 5). While this regulatory interaction and complex formation may occur as revealed in this study, further studies to discriminate between the presence of binary complexes from that of a ternary complex are required. Moreover,

The complexity of the mechanisms involved in SAC regulation highlights the importance of transcriptional regulation during SAC induction. Aside from PIGN, other proteins that previously had no physical interaction or functional association with the SAC such as WT1, RED, NUP153, YY1, SMURF2, TRRAP, and TAp73 have been reported to interact with and/or regulate the SAC^51–57^. However, tumor suppressors and proto-oncogenes have been shown to transcriptionally regulate SAC-related genes^58^. The loss of tumor suppressors like TP53, BRD4, and BRCA1 has been associated with altered expression of SAC-related genes, genomic instability, and tumor progression^59,60^. We have previously reported PIGN gene expression aberration-driven genomic instability in leukemic cells that was independent of the TP53–regulatory pathway^15^. Interestingly, TP53 has been shown in multiple studies to transcriptionally regulate the genes encoding MAD1, CDC20, cyclin A1 and cyclin B^59,61^. Moreover, the tumor suppressors, Rb, and VHL transcriptionally regulate the gene expression of the core SAC component MAD2^58,62^. Transcription factors like E2F, c-Myc, and FoxM1 and histone deacetylases have been linked to the transcriptional regulation of the SAC-related proteins including MAD2, PLK1 BUB3, and CDC20^58,60,61,63,64^. Yet, the possibility of the translocation of PIGN to the nucleus for the regulation of the transcription of cell cycle- or SAC-related genes may be complicated by the dissolution of the nuclear membrane during mitosis^65,66^. Thus, it will be necessary to experimentally determine whether PIGN regulates the SAC transcriptionally and/or translationally.

**Figure 6:**
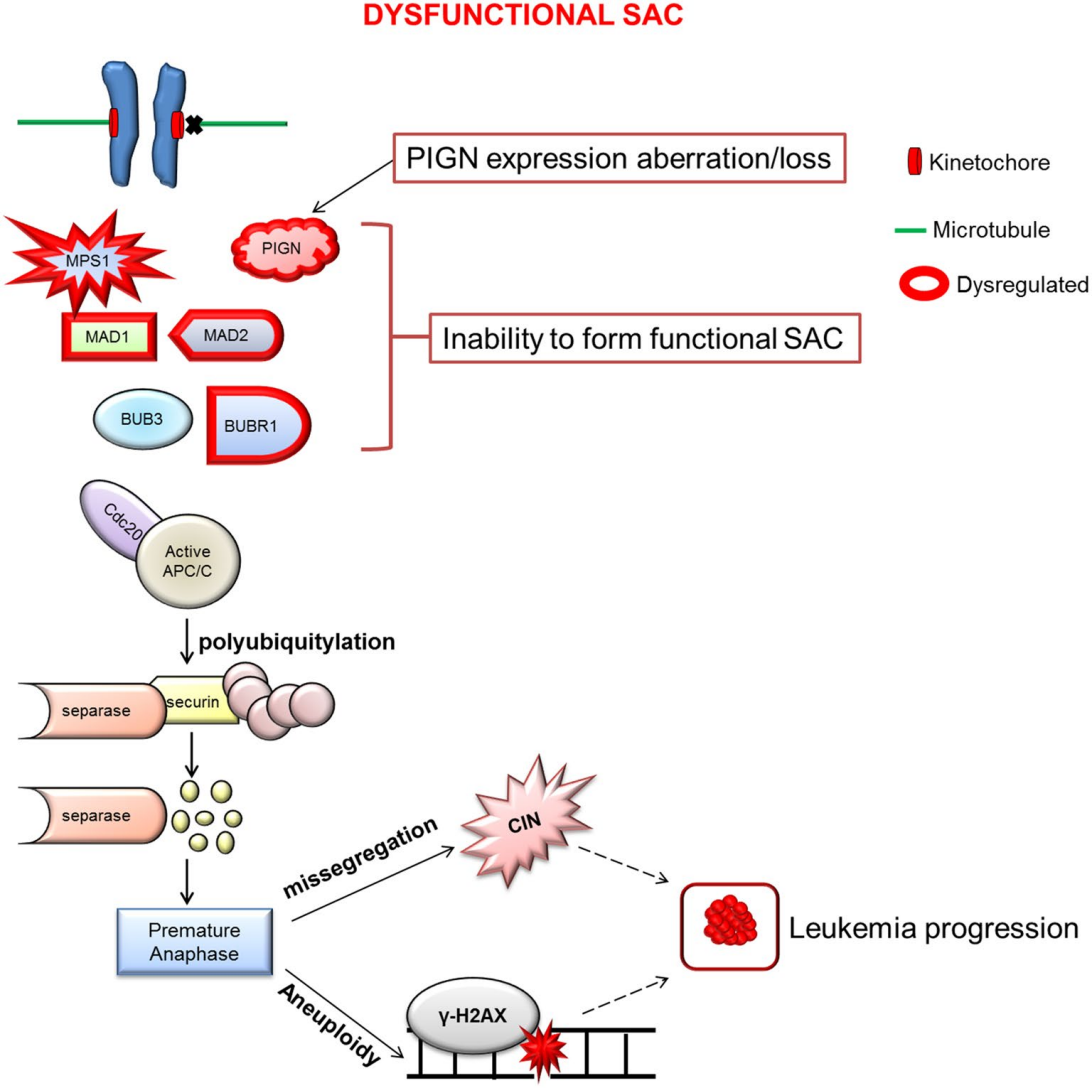
Model summarizing the relationship between PIGN expression aberration, SAC regulation, and leukemia progression. PIGN depletion results in SAC dysregulation and ultimately results in segregation errors and aneuploidy which contribute to leukemia progression. PIGN plays a vital role in the regulation of mitotic integrity to maintain chromosomal stability.

The ER membrane absorbs the nuclear membrane and dissolves into the cytosol which could explain the interaction between PIGN and MAD1 during mitosis despite the two proteins occupying separate cellular compartments^65–67^. Accordingly, nuclear proteins that are not normally localized at the ER may typically show up in the ER or cytosol during mitosis. However, it remains unclear how and where the interactions between PIGN and MAD1 occurs whether at the kinetochore, cytosol, or the ER. Although, the mitotic interaction between MAD1 and PIGN seemed to involve MAD2. PIGN may be involved in the stabilization of the interaction between MAD1 and MAD2, however, this requires experimental confirmation. The colocalization of the partial-intron retaining mutant of PIGN with MAD1 (Fig. 4D), implies that this intron-retaining mutant could compete with the wild-type protein for colocalization with these SAC proteins. Overall, PIGN maintains chromosomal stability, by interacting with and possibly regulating the SAC (Fig. 6).

## Conclusion

We identified PIGN as a novel SAC-interacting protein and potential regulator. The loss of PIGN impacts the expression of SAC signaling proteins and is linked to an accumulation of segregation errors, CIN, and ultimately leukemic transformation.

## Supplementary Information

Supplementary Information.
