# Correction: PIGN spatiotemporally regulates the spindle assembly checkpoint proteins in leukemia transformation and progression

**DOI:** 10.1038/s41598-026-48567-3

**Published:** 2026-06-03

**Authors:** Emmanuel K. Teye, Shasha Lu, Fangyuan Chen, Wenrui Yang, Thomas Abraham, Douglas B. Stairs, Hong-Gang Wang, Gregory S. Yochum, Robert A. Brodsky, Jeffrey J. Pu

**Affiliations:** 1 Penn State Cancer Institute, Pennsylvania State University College of Medicine, Hershey, PA USA; 2 Renji Hospital, Shanghai Jiao Tong University School of Medicine, Shanghai, China; 3 Institute of Hematology, Peking Union Medical College, Tianjin, China; 4 Division of Hematology, Johns Hopkins Medicine, Baltimore, MD USA; 5 University of Arizona Cancer Center, 1515 N Campbell Avenue, Tucson, AZ #1968C85724 USA

Correction to: *Scientific Reports* 10.1038/s41598-021-98218-y, published online 24 September 2021

This Article contains errors.

Several of the immunoblot and cell cycle synchronization panels were derived from the same experimental immunoblot films or flow cytometry datasets as described in our previous work, listed below as Ref.^1^. These datasets were reused as reference data to support mechanistic analyses of spindle assembly checkpoint (SAC) regulation by PIGN, but the re-use is not disclosed in this Article. This should have been disclosed in the legends of Figures 1–3. Therefore, Figure 1 legend:

“PIGN expression is cell cycle regulated. (A) Schematic of cell cycle synchronization protocols. Cell synchronizations were achieved using serum starvation for 72 h (Go/G1), double-thymidine block (G1/S), or double thymidine block and release followed by nocodazole treatment (G2/M). Cells were collected post-treatment for western blotting, RT-qPCR, and flow cytometry analyses. PIGN was expressed in a cell cycle-dependent manner in multiple cell lines: (B) HL60, (C) K562, (D) KCL22, and (E) JURKAT with suppressed expression from early S-phase to the G2/M phase. All western blot images were cut within the molecular weight ranges of the protein targets prior to hybridization. Images generated from these cut blots by scanning the developed films. Images were cropped and labeled using Adobe Photoshop CC 2017 (version 18). Where applicable, representative full images of the blots are presented in Figure 3 of the supplementary immunoblotting data.”

should read:

“PIGN expression is cell cycle regulated. (**A**) Schematic of cell cycle synchronization protocols. Cell synchronizations were achieved using serum starvation for 72 h (Go/G1), double-thymidine block (G1/S), or double thymidine block and release followed by nocodazole treatment (G2/M). Cells were collected post-treatment for western blotting, RT-qPCR, and flow cytometry analyses. PIGN was expressed in a cell cycle-dependent manner in multiple cell lines: (**B**) HL60, (**C**) K562, (**D**) KCL22, and (**E**) JURKAT with suppressed expression from early S-phase to the G2/M phase. All western blot images were cut within the molecular weight ranges of the protein targets prior to hybridization. Images generated from these cut blots by scanning the developed films. Images were cropped and labeled using Adobe Photoshop CC 2017 (version 18). Where applicable, representative full images of the blots are presented in Figure 3 of the supplementary immunoblotting data. The flow cytometry histograms and corresponding PIGN and MAD1 immunoblots shown in panels B and C (HL60 and K562) were derived from the same flow cytometry acquisition runs generated during the original cell cycle synchronization experiments reported in^1^, (Figure 7A). These panels are presented here as validated reference data to enable direct comparison with additional cell lines (KCL22 and Jurkat).”

Figure 2 legend,

“PIGN loss or suppression results in the differentially disrupted expression of SAC components. (**A**) CRISPR/Cas9 ablation of PIGN led to MAD1 and MAD2 downregulation in CD34+ mononuclear cells from a healthy donor. (**B**) Gene and protein expressions of MAD1 and MAD2 were significantly (***p < 0.0001) impacted by PIGN loss in CD34+ mononuclear cells from a healthy donor. (**C,D**) Complete loss of PIGN via CRISPR/Cas9 ablation (KO) was associated with downregulation of MAD1, MAD2, and MPS1 while causing an upregulated gene (***p < 0.0001) and protein expression of BUBR1 in HEK293 cells. PIGN loss resulted in significant repression (*p< 0.05) of MAD1 and MAD2 gene expression but led to MPS1 gene upregulation (*p < 0.05). (**E**) PIGN loss (KO) increased the frequency of segregation errors in HEK293 cells. (**F**) Ectopic overexpression of PIGN restored MAD2 expression in HEK293 PIGN KO cells. (**G**) RNAi-mediated PIGN suppression resulted in MAD1 and MAD2 downregulation but increased expression of BUBR1 and MPS1 expression in K562 cells. (**H**) MAD1 suppression was accompanied by a corresponding decrease in PIGN protein expression in K562 cells. (**I**) CRISPR/Cas9 ablation of PIGN (K562 KO) resulted in MAD1, BUBR1, and MPS1 downregulation in K562 cells. (**J**) RNAi-mediated MAD1 suppression in K562 cells resulted in MAD1 downregulation while causing an upregulation in BUbR1 and MPS1 expression. (K) AML-MRC patient CD34+ PBMCs (P1 and P2) with PIGN partial intron retentions showed a significant (***p < 0.0001) increase in PIGN and MAD1 gene expressions compared to non-leukemic control cells from a healthy donor. All western blot images were cut within the molecular weight ranges of the protein targets prior to hybridization. Images generated from these cut blots by scanning the developed films. Images were cropped and labeled using Adobe Photoshop CC 2017 (version 18). Where applicable, representative full images of the blots are presented in Figure 2 of the supplementary immunoblotting data.”

should read:

“PIGN loss or suppression results in the differentially disrupted expression of SAC components. (**A**) CRISPR/Cas9 ablation of PIGN led to MAD1 and MAD2 downregulation in CD34+ mononuclear cells from a healthy donor. (**B**) Gene and protein expressions of MAD1 and MAD2 were significantly (***p < 0.0001) impacted by PIGN loss in CD34+ mononuclear cells from a healthy donor. (**C,D)**. Complete loss of PIGN via CRISPR/Cas9 ablation (KO) was associated with downregulation of MAD1, MAD2, and MPS1 while causing an upregulated gene (***p < 0.0001) and protein expression of BUBR1 in HEK293 cells. PIGN loss resulted in significant repression (*p < 0.05) of MAD1 and MAD2 gene expression but led to MPS1 gene upregulation (* *p* < 0.05). (E) PIGN loss (KO) increased the frequency of segregation errors in HEK293 cells. (**F**) Ectopic overexpression of PIGN restored MAD2 expression in HEK293 PIGN KO cells. (**G**) RNAi-mediated PIGN suppression resulted in MAD1 and MAD2 downregulation but increased expression of BUBR1 and MPS1 expression in K562 cells. (**H**) MAD1 suppression was accompanied by a corresponding decrease in PIGN protein expression in K562 cells. (**I**) CRISPR/Cas9 ablation of PIGN (K562 KO) resulted in MAD1, BUBR1, and MPS1 downregulation in K562 cells. (**J**) RNAi-mediated MAD1 suppression in K562 cells resulted in MAD1 downregulation while causing an upregulation in BUbR1 and MPS1 expression. (**K**) AML-MRC patient CD34+ PBMCs (P1 and P2) with PIGN partial intron retentions showed a significant (***p < 0.0001) increase in PIGN and MAD1 gene expressions compared to non-leukemic control cells from a healthy donor. All western blot images were cut within the molecular weight ranges of the protein targets prior to hybridization. Images generated from these cut blots by scanning the developed films. Images were cropped and labeled using Adobe Photoshop CC 2017 (version 18). Where applicable, representative full images of the blots are presented in Figure 2 of the supplementary immunoblotting data. Selected immunoblot lanes in panels C-J derive from the same CRISPR/Cas9 and RNAi-mediated PIGN perturbation experiment series previously used in^1^ (2017, Figure 7B-G). In this Article, these datasets were reanalyzed to expand SAC pathway mapping to additional components (MAD2, BUBR1, MPS1), incorporate rescue experiments via PIGN re-expression, and provide quantitative segregation error analyses. Blots were prepared from the same perturbation batches.”

and finally Figure 3 legend:

“PIGN forms a complex with SAC components. MAD1 and MAD2 were co-purified with PIGN in an HA-tag pull-down assay in PIGN null HEK293 cells ectopically expressing 3HA-tagged PIGN. (**A,B**) HA-tag IP in asynchronous cells indicated optimal 3HA-tagged PIGN expression and co-purification with MAD1 and MAD2 at the 48-h time point. (**C**) MAD1 and MAD2 were co-purified with PIGN in G2/M synchronized cells. (**D**) Co-immunoprecipitation (Co-IP) experiment showed that PIGN endogenously interacted with MAD1 and MAD2 during SAC activation via Taxol (60 ng/µl) or nocodazole (60–100 ng/µl) treatment of K562 cells for 12 h. (**E**) HA-tag pull-down assay with cells released from early S-phase into mitosis indicated PIGN co-purification with MAD1 and MPS1. Inputs represent 5–10% of total protein lysate. All western blot images were cut within the molecular weight ranges of the protein targets prior to hybridization. Images generated from these cut blots by scanning the developed films. Images were cropped and labeled using Adobe Photoshop CC 2017 (version 18). Where applicable, representative full images of the blots are presented in Figure 3 of the supplementary immunoblotting data.”

should read:

“PIGN forms a complex with SAC components. MAD1 and MAD2 were co-purified with PIGN in an HA-tag pull-down assay in PIGN null HEK293 cells ectopically expressing 3HA-tagged PIGN. (**A,B**) HA-tag IP in asynchronous cells indicated optimal 3HA-tagged PIGN expression and co-purification with MAD1 and MAD2 at the 48-h time point. (**C**) MAD1 and MAD2 were co-purified with PIGN in G2/M synchronized cells. (**D**) Co-immunoprecipitation (Co-IP) experiment showed that PIGN endogenously interacted with MAD1 and MAD2 during SAC activation via Taxol (60 ng/µl) or nocodazole (60–100 ng/µl) treatment of K562 cells for 12 h. (**E**) HA-tag pull-down assay with cells released from early S-phase into mitosis indicated PIGN co-purification with MAD1 and MPS1. Inputs represent 5–10% of total protein lysate. All western blot images were cut within the molecular weight ranges of the protein targets prior to hybridization. Images generated from these cut blots by scanning the developed films. Images were cropped and labeled using Adobe Photoshop CC 2017 (version 18). Where applicable, representative full images of the blots are presented in Figure 3 of the supplementary immunoblotting data. Panels A-C derive from the same HA-tag PIGN pulldown and co-immunoprecipitation film series originally generated and partially displayed in^1^ (2017, Figure 7H). In the current study, these data are presented to assess cell cycle timing, mitotic synchronization (G2/M), and expanded SAC interaction partners (MAD1, MAD2, MPS1). The reuse reflects reanalysis of the same experimental series to address the spatiotemporal context not examined in the earlier publication.”
