# Supplementary material for: PIGN spatiotemporally regulates the spindle assembly checkpoint proteins in leukemia transformation and progression
Source: Sci Rep. 2021 Sep 24;11:19022. doi: 10.1038/s41598-021-98218-y (PMC8463542; doi:10.1038/s41598-021-98218-y)
Supplement: Supplementary file 1 — Supplementary Information. [file 41598_2021_98218_MOESM1_ESM.docx]

1. **Article Title**:

PIGN Spatiotemporally Regulates the Spindle Assembly Checkpoint Proteins in Leukemia Transformation and Progression

**2)** **Short Title**:

PIGN Regulates the Spindle Assembly Checkpoint Proteins in Leukemia

3) **Author List**:

Emmanuel K. Teye ^1^, Shasha Lu ^1,2^, Fangyuan Chen^2^, Wenrui Yang ^1,3^, Thomas Abraham ^1^, Douglas B. Stairs ^1^, Hong-Gang Wang ^1^, Gregory S. Yochum ^1^, Robert A. Brodsky^4^, Jeffrey J. Pu ^1,5*^

**4) Affiliate Institutions**:

1. Penn State Cancer Institute, Pennsylvania State University College of Medicine, Hershey, Pennsylvania, USA

2. Renji Hospital, Shanghai Jiao Tong University School of Medicine, Shanghai, China

3. Institute of Hematology, Peking Union Medical College, Tianjin, China

4. Division of Hematology, Johns Hopkins Medicine, Baltimore, Maryland, USA

5. University of Arizona Cancer Center, Tucson, Arizona, USA

**5) Corresponding Author***:

Jeffrey J. Pu, MD, Ph.D.

#1968C, 1515 N Campbell Avenue, Tucson, Arizona 85724

Phone: 520-626-0662, Fax: 520-626-2225

Email: [jeffreypu@gmail.com](mailto:jeffreypu@gmail.com)

**Supplementary Materials and Method**

***Cell culture***

Leukemia and lymphoblastoid cell lines HL60, K562, Jurkat, KCL22, KG1, and KG1a cells were cultured at 37⁰C 5% CO_2_ with RPMI or IMDM supplemented with 20% FBS. HEK293 and HEK293 PIGN CRISPR/Cas9 Knockout cells were grown in DMEM supplemented with 10% FBS. Cell lines were passaged on average every 3-4 days. As previously described, CD34+ mononuclear cells were isolated from the peripheral blood of a healthy volunteer and cultured in DMEM/F12 supplemented with 10% FBS, 50 μM 2-mercaptoethanol, Glutamax (Life Technologies), MEM non-essential amino acids (Life Technologies) and StemMACS HSC expansion cocktail (Miltenyi Biotec)^18^. All cells were cultured at 37°C under 5% CO_2_ conditions. All cell lines were conducted short tandem repeat (STR) profile analysis using Geneprint 10 System Kit (Promega, Madison, WI, USA) and compared to know ATCC fingerprints (ATCC.org) for authentication every 3 months. Mononuclear cells were isolated from the blood or bone marrow aspirates of donors using the Ficoll-Paque PLUS reagent (GE Healthcare) according to the manufacturer’s protocol.

***Gene expression analyses***

RT-qPCR experiments were conducted as described previously [1]. Total RNA was isolated from the cells using the RNeasy Mini Kit (Qiagen) according to the manufacturer’s protocol. Total RNA was reverse transcribed using the High Capacity cDNA reverse transcription kit (Life Technologies) on the Mastercycler Nexus (Eppendorf). The RT-qPCR step was conducted on the StepOnePlus real-time PCR System (Applied Biosystems) using *PIGN* (Hs00202443_m1), *MAD1* (Hs00269119_m1) and *MAD2* (Hs01554513_g1) gene expression assay (Life Technologies) and 18S (rRNA Hs99999901_s1) (Life Technologies) gene expression as an internal reference control. *BUBR1* and *MPS1* primers as well as *GAPDH* internal control primers (Integrated DNA Technologies) (see Table 1 for primer sequences) were used with Power SYBR Green PCR Master Mix (Life Technologies). For all experiments, samples were run in triplicates, and expression data were normalized to *PIGN* gene expression in the control group. Gene expression fold changes were calculated using the ΔΔCt method.

***HA-tag immunoprecipitation (IP)/ and co-immunoprecipitation (Co-IP) analyses***

PIGN-HA IP/Co-IP experiments were conducted by transient transfection of CRISPR/Cas9 PIGN knockout HEK293 cells with the SRα promoter-driven expression vector pMEPuro3HAhPIGN or the empty vector without PIGN cDNA cloned [2]. HEK293 CRISPR KO cells and pMEPURO3HAhPIGN expression plasmids were the gifts from Drs. Taroh Kinoshita and Yoshiko Murakami, Research Institute for Microbial Diseases, Osaka University. The cells were transfected with 2.5-5 μg of the vector using the Lipofectamine 3000 transfection reagent (Life Technologies) according to the manufacturer’s protocol. Protein samples were evaluated 24-72 hours post-transfection, and 250-500 μg protein was used with the HA-tag IP/Co-IP kit according to the manufacturer's protocol. The eluates and 10% of the input lysates were used for Western blot analyses. Co-IP experiments were conducted with the Pierce Co-IP kit per the manufacturer’s protocol. Before the co-IP experiments, the cells were treated with 60 ng/µl Taxol (Bristol-Myers Squibb) and 60-100 ng/µl Nocodazole (Sigma) for 12 hours to activate the SAC. For each experiment 2 mg of the whole-cell lysate was used and 20% of the lysate sample was loaded for use as input control. The MAD1 antibody (GTX109519, GeneTex) antibody was employed for the Co-IP experiments.

***Western blot analyses***

Western blot analysis was conducted as described previously [1]. Briefly, total protein were extracted using RIPA cell lysis buffer supplemented with phosphatase inhibitor cocktail and protease inhibitor (Sigma). Whole-cell lysates were subjected to electrophoresis in a NuPAGE 4-12% or 10% Bis-Tris Gel (Life Technologies) and transferred to an Immun-Blot PVDF Membrane (Bio-Rad). The membranes were blocked in 5% milk in TBS-T (i.e. 0.1% v/v Tween 20) for 1 hour at room temperature and subsequently treated with primary antibodies (1:500-1:1000) prepared in 5% milk/TBS-T overnight at 4ºC. The blots were incubated overnight with anti-PIGN antibodies (HPA039922, Atlas Antibodies) anti-MAD1 antibodies (Clone BB3-8, MABE867, Millipore), anti-Histone H2A.X (D17A3, Cell Signaling Technology) anti-phospho-Histone H2A.X ser139 (S139, Cell Signaling Technology), cyclin B1 (4138, Cell Signaling Technology) and HA-Tag(C29F4, Cell Signaling Technology) in TBS-T with 5% Non-Fat Dry Milk. Mouse beta-actin (C-4, sc-47778, Santa Cruz Biotechnology) was used as the loading control. The membranes were then washed three times with TBS-T and incubated for 1-2 hours with a horseradish peroxidase-conjugated goat anti-rabbit (AP132P) or goat anti-mouse (AP124P) IgG secondary antibody (1:5000-1:10000; Millipore). Afterward, membranes were washed three times with TBS-T and treated with ECL Prime Western Blotting detection reagents (Amersham) to detect proteins. Images were acquired using Image Lab Software for PC Version 6.1 (Bio-Rad).

***PIGN knockdown and CRISPR/Cas9 Knockout studies***

RNAi-mediated *PIGN* knockdown experiments were conducted using the Nucleofector II Device (Amaxa) in conjunction with the Cell line Nucleofector Kit V reagent kit (Amaxa). CRISPR/Cas9 experiments were performed according to a modified LentiCRISPRv2 (Addgene plasmid #49535) protocol [3]. The gRNA (AAACGGTCATGTAGCTCTGATAGC) we employed targets *PIGN* at exon 4 and results in a frameshift [2]. Lentiviral-transduced CD34+ mononuclear cells were harvested for downstream applications nine days post-infection according to a modified protocol [4]. PIGN knockdown and CRISPR/Cas9 Knockout studies RNAi-mediated PIGN knockdown experiments were conducted using the Nucleofector II Device (Amaxa) in conjunction with the Cell line Nucleofector Kit V reagent kit (Amaxa) by the manufacturer’s recommended protocols for the respective cell lines. The cells were transfected with 100 nM siGENOME siRNA Human PIGN, (D-012463-01, Dharmacon), ON-TARGETplus Human MAD1L1 (8379) siRNA-SMARTpool (L-006825-00-0005, Dharmacon) or 100 nM siGENOME Control siRNA non-targeting siRNA #2, (D-001210-02-05, Dharmacon) and incubated for 24-72 hours.

***Cell cycle analyses and SAC activation***

Cell cycle synchronizations were performed as reported previously [5]. Serum starvation for 72 hours was used to synchronize cells in G0 as described previously [5]. Cells were synchronized at the Go/G1, S, and G2/M phases (Figure 1A). Cell cycle synchrony was monitored using propidium iodide-stained cells with the BD FACSCalibur flow cytometer (BD Biosciences). Protein lysates were obtained and used for Western blot analyses as described previously [1].

***Immunofluorescence and confocal microscopy***

For missegregation and colocalization analyses, cells were blocked in early S-phase via the double-thymidine treatment and released for 6-8 hours into the mitotic phase. Adherent cells were cultured on chambered slides. Cytospin was used to fix suspension cells onto the slides. Cells were fixed in 4% paraformaldehyde in PBS and permeabilized in -20°C 100% methanol. The slides were blocked with 2.5% normal goat serum diluted in PBS. For all wash steps, the cells were washed three times for 5 minutes each in PBS. The cells were treated at 4°C overnight with primary antibodies: Human anti-centromere (kinetochore) (15-234, Antibodies Incorporated), PIGN (HPA039922, Atlas Antibodies), MAD1 (Clone BB3-8, MABE867, Millipore), MAD2 (sc-374131, Santa Cruz), BUBR1 (720297, ThermoFisher) or MPS1 (05-682, Millipore) followed by washing and treatment for 2 hours at room temperature in the dark with secondary antibodies (1:1000): Alexa Fluor 647 goat anti-human IgG (H+L) (A21445, Life Technologies), Goat anti-rabbit IgG, Dylight 488 Conjugated Highly cross-adsorbed (35553, Life Technologies) or Goat anti-mouse IgG, Dylight 633 Conjugated (35513, Life Technologies) respectively. Antibodies were diluted in 1% normal goat serum diluted in PBS. The slides were partially dried in the dark and mounted in Vectashield Hard Set mounting medium with DAPI (H-1500, Vector Laboratories). Images were acquired using the Leica SP8 inverted confocal microscope at the Microscopy Imaging Facility at Penn State College of Medicine. Three-dimensional image stacks were acquired in 0.15-μm steps using a ×40 1.4 N.A oil immersion objective. Deconvolution and analyses of image stacks were performed using the Huygens workstation (Scientific Volume Imaging B.V.), the Imaris Microscopy Image Analysis (Bitplane AG) and Volocity 6.3 (PerkinElmer Inc).

***Mitotic index and cell cycle frequency analyses***

HEK293 cells were detached from the plate tapping the sides of the plate and very gently scraping. The detached cells were washed with PBS once and passed through a cell filter to obtain single cells and remove clumps. Some of the cells in each treatment group were reserved for use as H3 and PI staining positive controls and as unstained controls. The cells were centrifuged at 2000 rpm for 5 min and resuspended in 150 μl of PBS and 350 μl ethanol and kept on ice for at least 1 hour. Then ethanol was then removed by centrifugation of the cells and the cells were resuspended in PBS containing 0.25% Triton X-100 (10 ml PBS+ 25 μl Triton X-100) and again incubated on ice for 15 minutes. Post-incubation, the supernatant was removed, and the cells were resuspended in 100 μl of PBS containing 1% BSA and 0.25 μg of anti-phospho-Histone H3 (Ser10) (05-806, Millipore Sigma) and incubated for 1 h at room temperature. The cells were spun down and washed with 150 μl of PBS containing 1% BSA. The cells were afterward resuspended in Alexa 488-conjugated goat anti-mouse immunoglobulin G antibody (A32723, ThermoFisher Scientific) (1:300) in 100 μl of PBS containing 1% BSA and incubated at room temperature in the dark for at least 30 minutes. After incubation, the cells were centrifuged and resuspended in PI/ RNase A, transferred to FACS tubes and incubated at room temperature in the dark for at least 30 minutes after which flow cytometry analyses were conducted on a 2-color AlexaFluor488 (FITC) vs PI or stored at 4 °C until FACS analysis.

***Statistical analyses***

GraphPad Prism 8 and Microsoft Excel were used for statistical analyses. Two-tailed Student’s t-tests, one-way or two-way ANOVA followed by Tukey’s post hoc tests for multiple comparisons; p-values ≤ 0.05 were considered statistically significant.

**Reference**

1. Teye EK, Sido A, Xin P, et al. PIGN gene expression aberration is associated with genomic instability and leukemic progression in acute myeloid leukemia with myelodysplastic features. *Oncotarget*. 2017;8(18):29887–29905.

2. Ohba C, Okamoto N, Murakami Y, et al. PIGN mutations cause congenital anomalies, developmental delay, hypotonia, epilepsy, and progressive cerebellar atrophy. *Neurogenetics*. 2014;15(2):85–92.

3. Sanjana NE, Shalem O, Zhang F. Improved vectors and genome-wide libraries for CRISPR screening. *Nat. Methods*. 2014;11(8):783–784.

4. Mandal PK, Ferreira LMR, Collins R, et al. Efficient ablation of genes in human hematopoietic stem and effector cells using CRISPR/Cas9. *Cell Stem Cell*. 2014;15(5):643–652.

5. Jackman J, O’Connor PM. Methods for synchronizing cells at specific stages of the cell cycle. *Curr. Protoc. Cell Biol.* 2001; Chapter 8:Unit 8.3

**Supplementary immunoblotting images**

**Figure 1**

**Figure 1b**


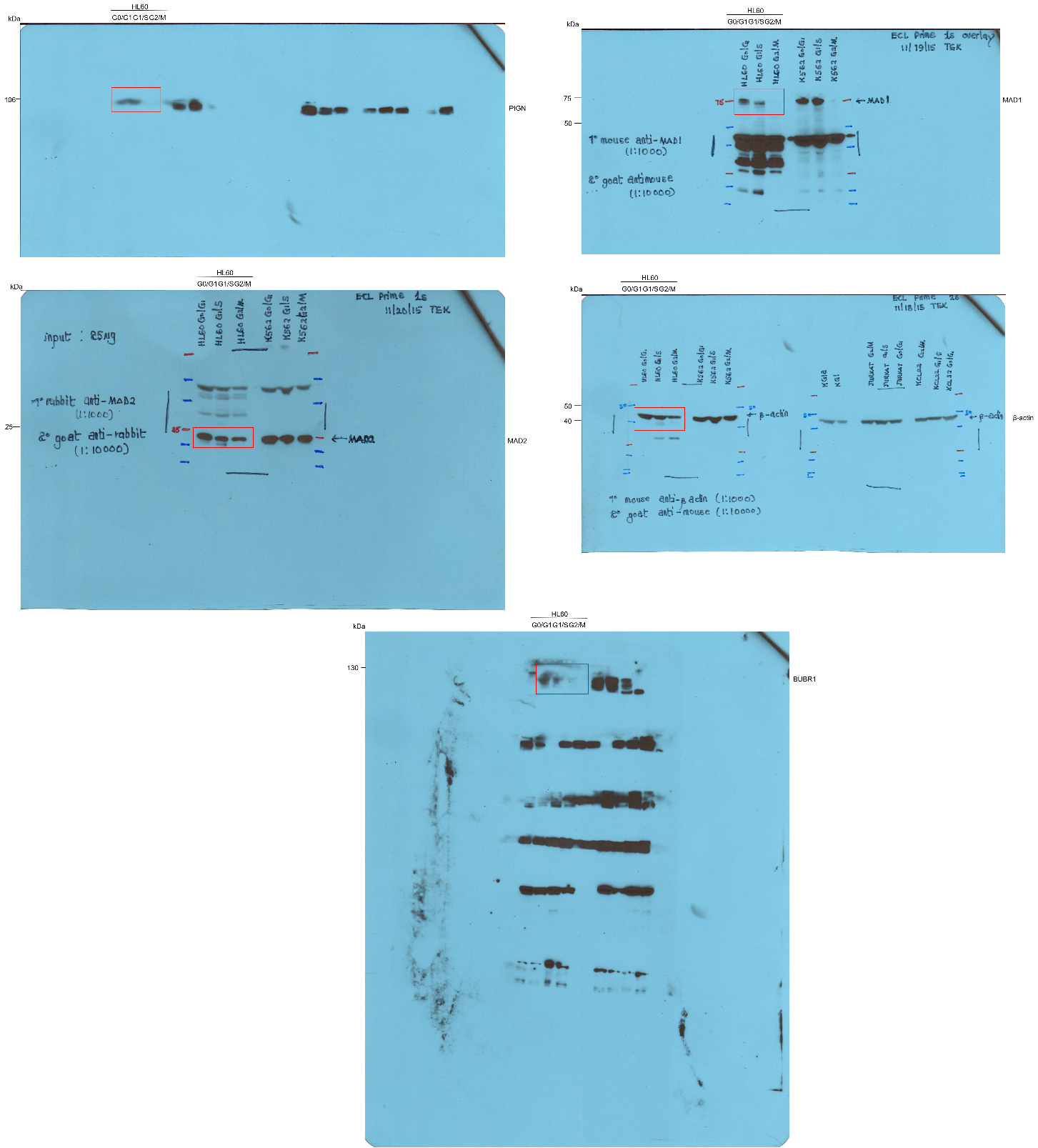


**Figure 1c**


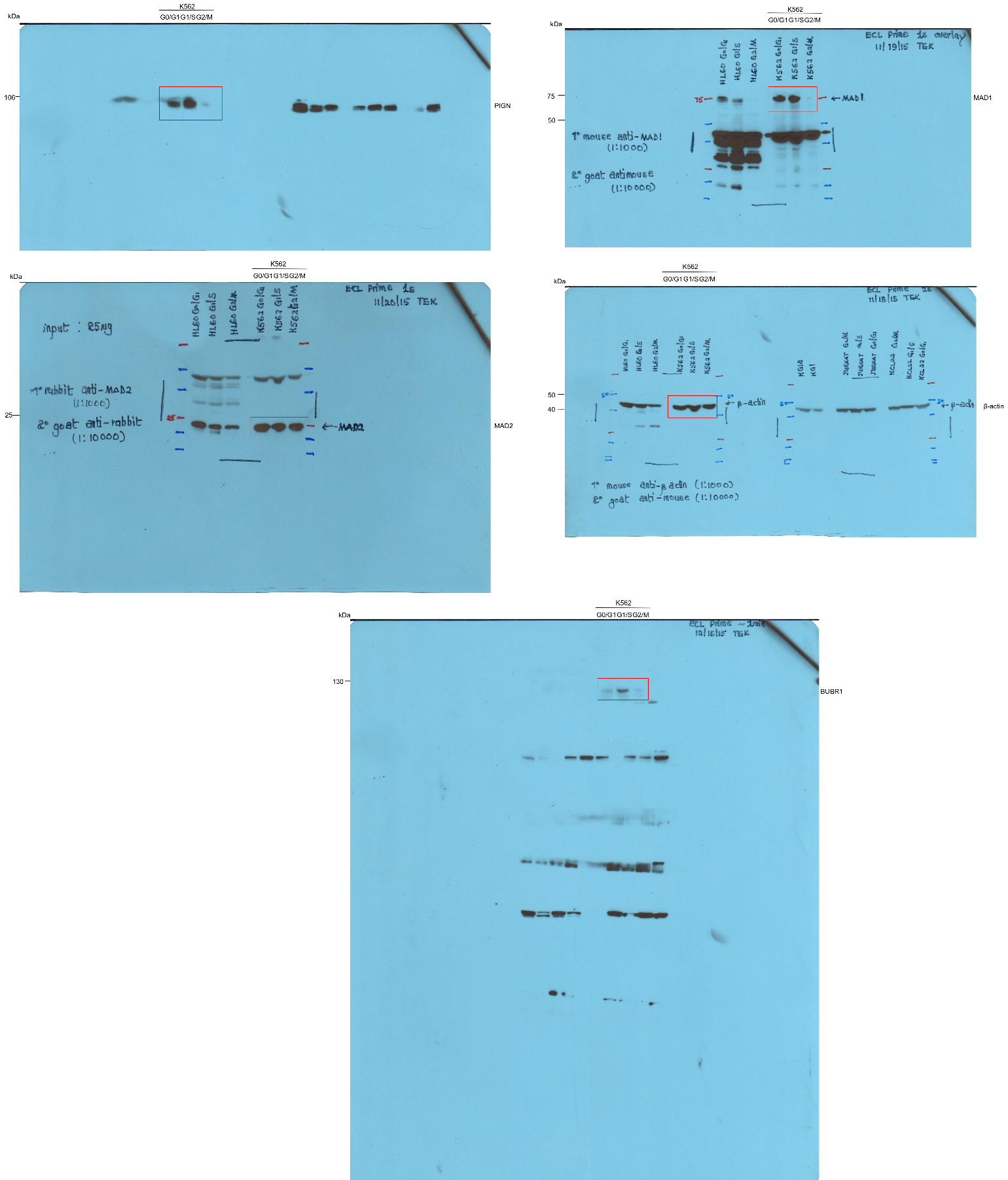


**Figure 1d**


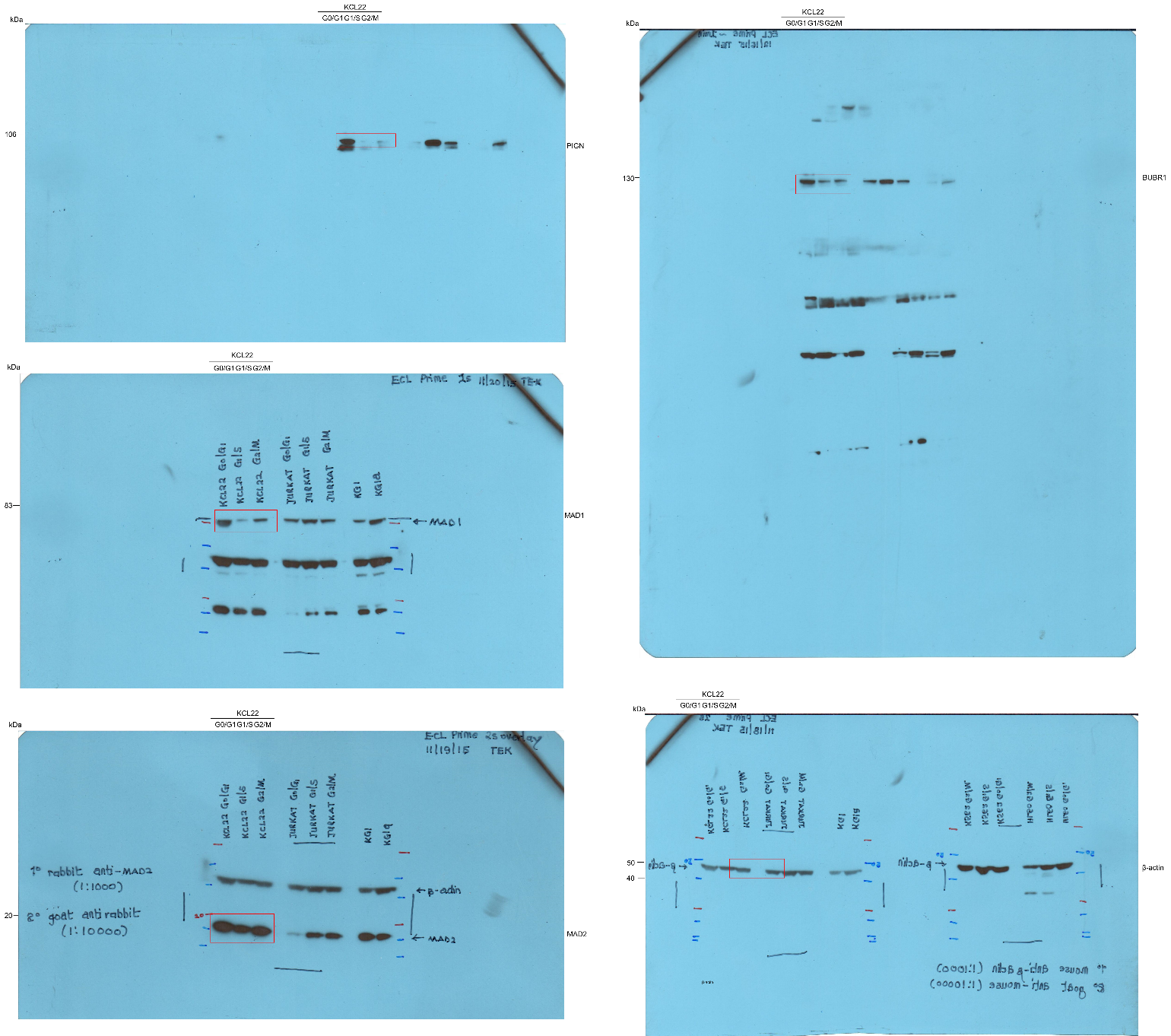


**Figure 1e**


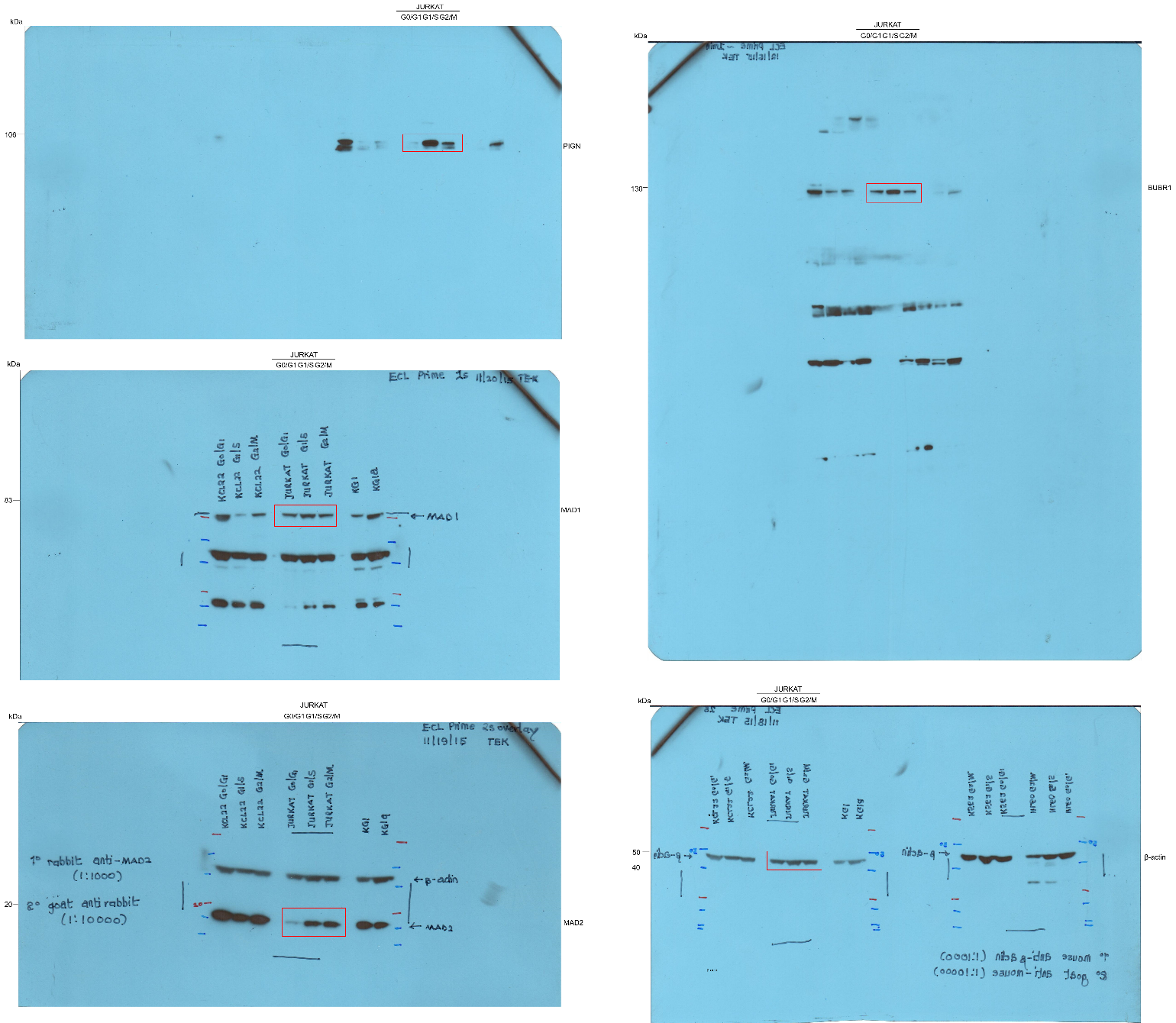


**Figure 2**

**Figure 2a**


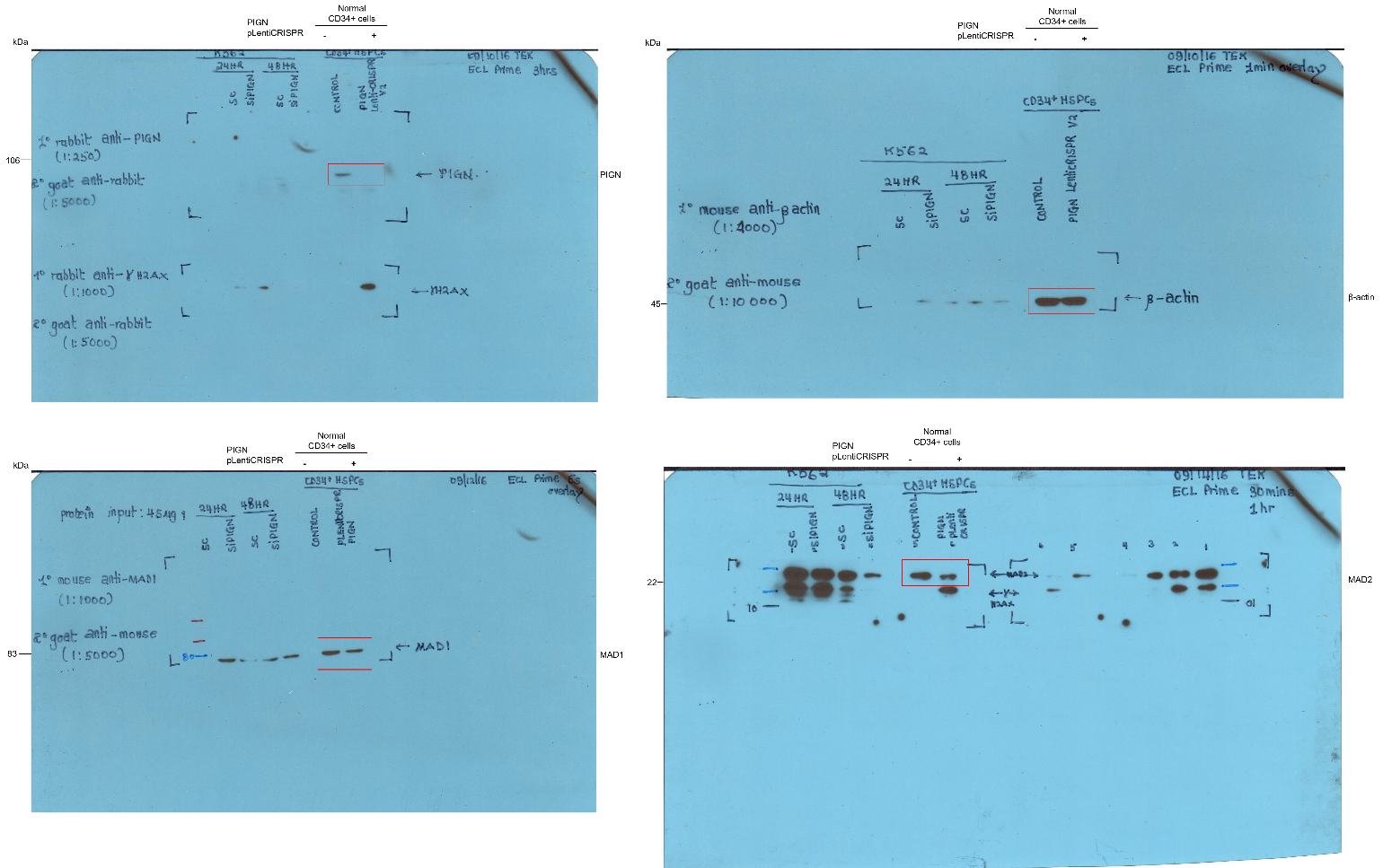


**Figure 2c**


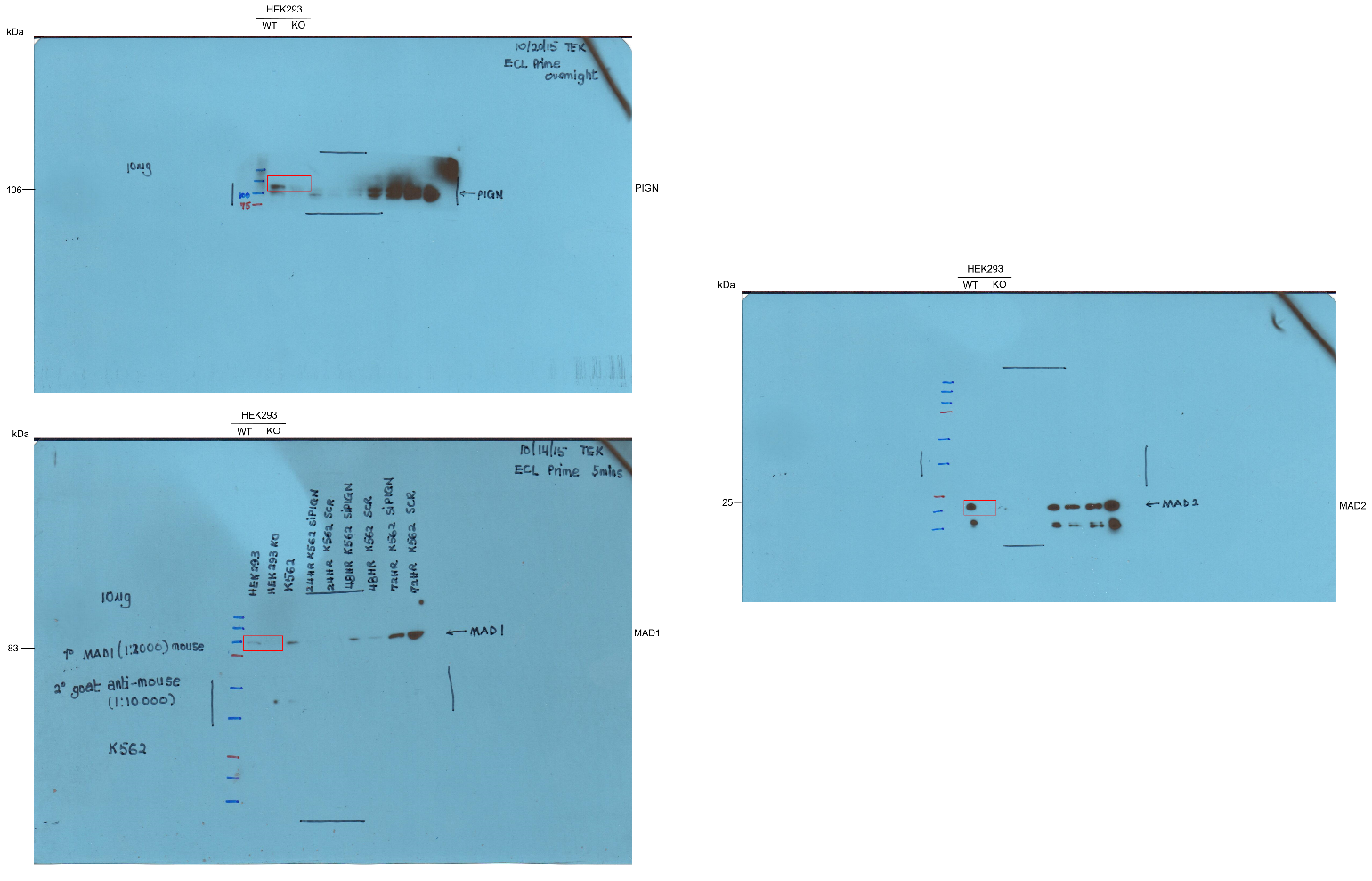


Full blot images are not presented for BubR1, MPS1 and β-actin in Figure 2c; representative blots shown in supplementary immunoblotting Figures 1a (BubR1 and β-actin) and 3e (MPS1).

**Figure 2f**


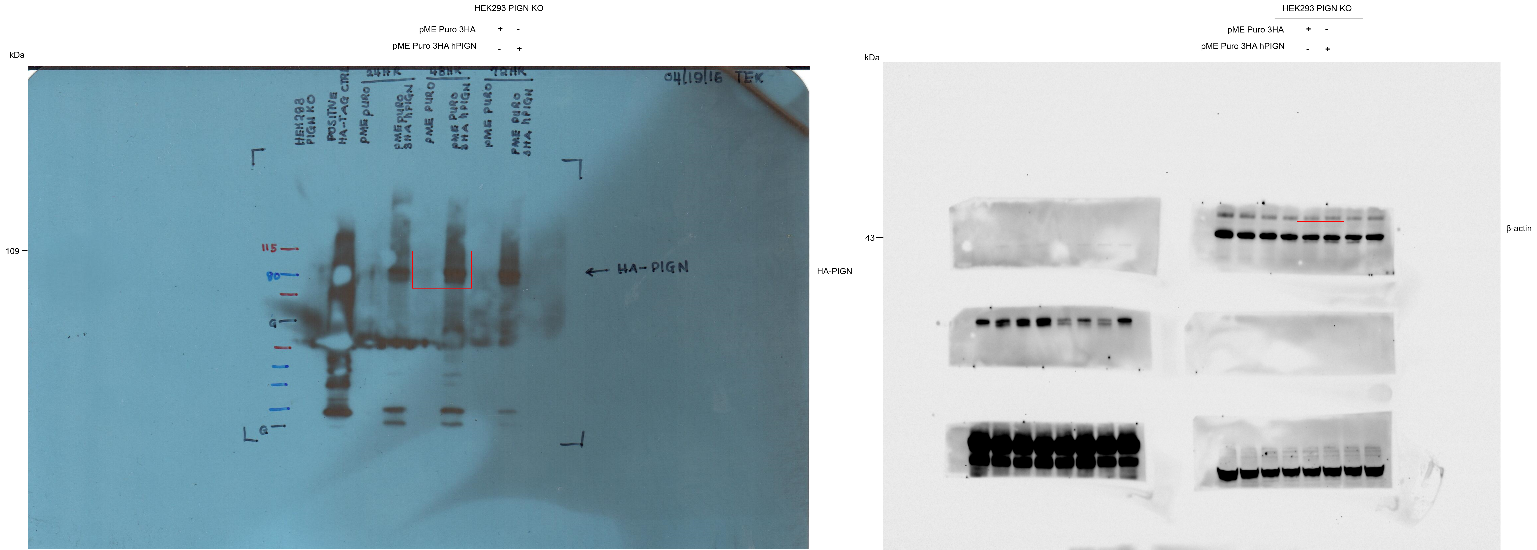


Full blot images are not presented for MAD2. Representative full film shown in supplementary immunoblot Figure 2a

**Figure 2g**


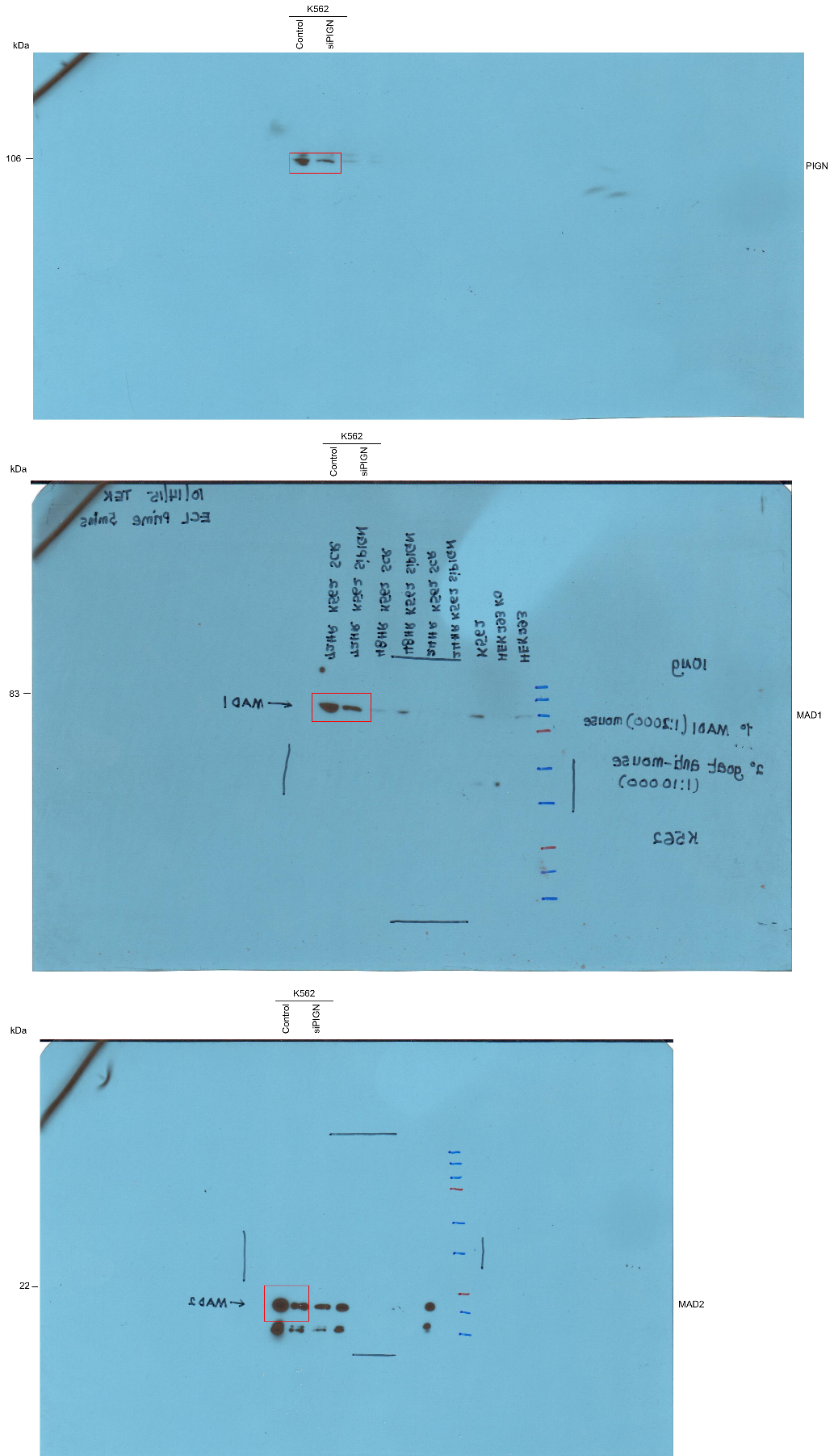


Full blot images are not presented for BubR1, MPS1 and β-actin in Figure 2c; representative blots shown in supplementary immunoblotting Figures 1a (BubR1 and β-actin) and 3e (MPS1).

**Figure 2i**

Full blot images are not presented for PIGN, MAD1, MAD2, BubR1, MPS1 and β-actin in Figure 2i; representative blots shown in supplementary immunoblotting Figures 2c using the same antibodies as indicated in the Western blot supplementary materials sections.

**Figure 2j**


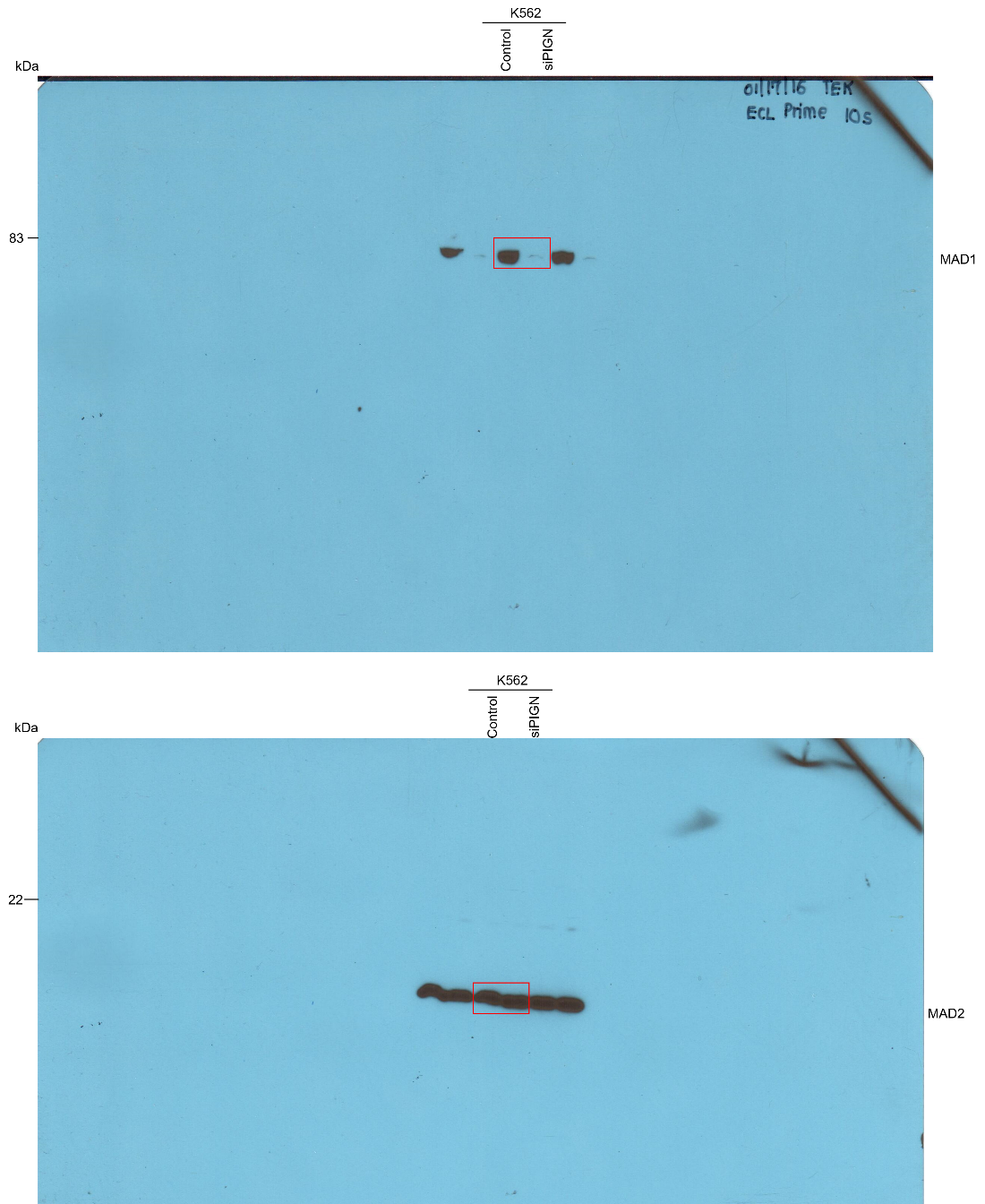


Full blot images are not presented for PIGN, BubR1, MPS1 and β-actin in Figure 2i; representative blots shown in supplementary immunoblotting Figures 2c using the same antibodies as indicated in the Western blot supplementary materials sections

**Figure 3**

**Figure 3a**


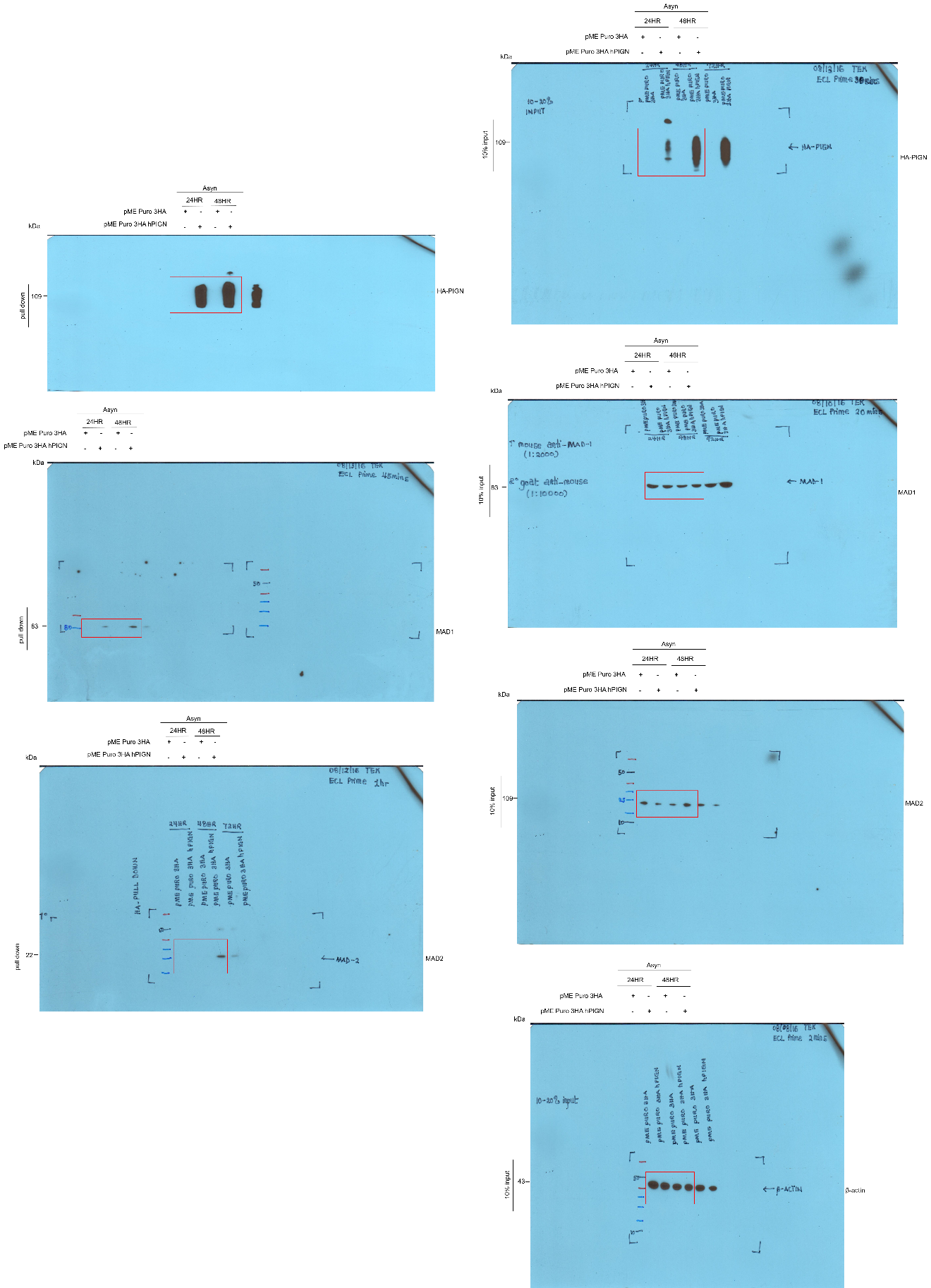


**Figure 3b**


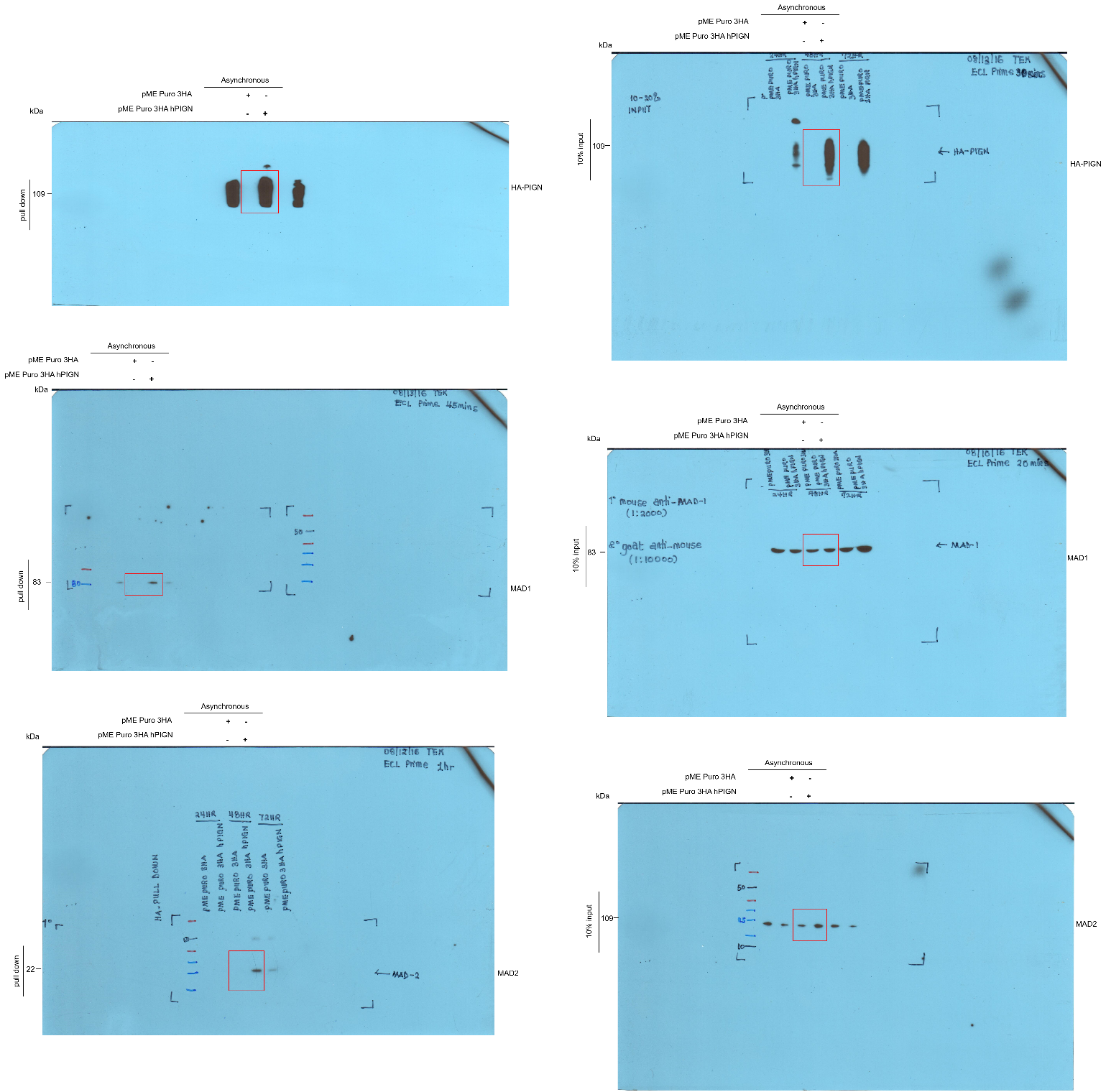


**Figure 3c**


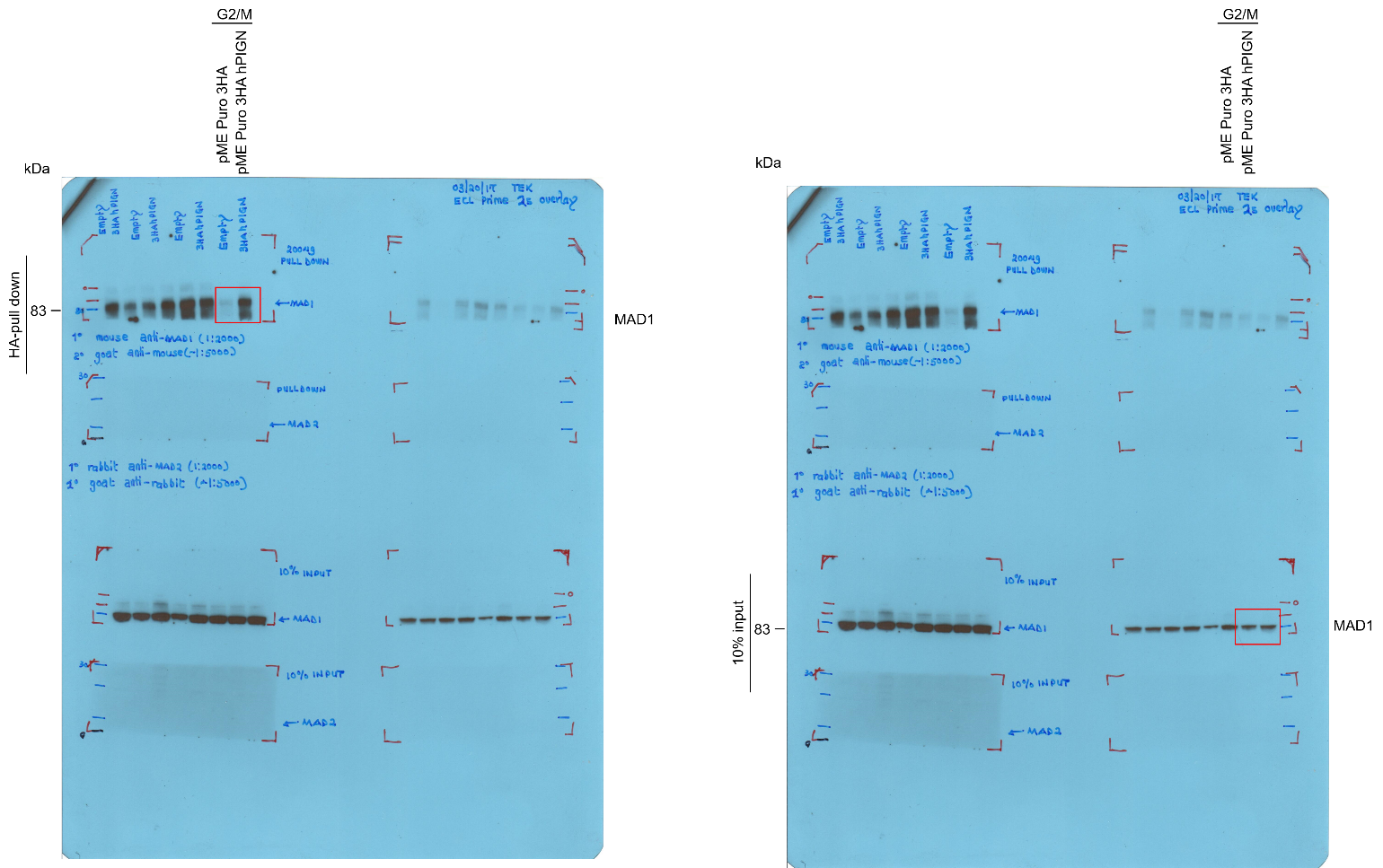


Full blot images are not presented for HA-PIGN and MAD2 (HA-pulldown and 10% input); representative blots shown in supplementary immunoblotting Figures 3a and b which were developed using the same antibodies as indicated in the Western blot supplementary materials section.

**Figure 3d**


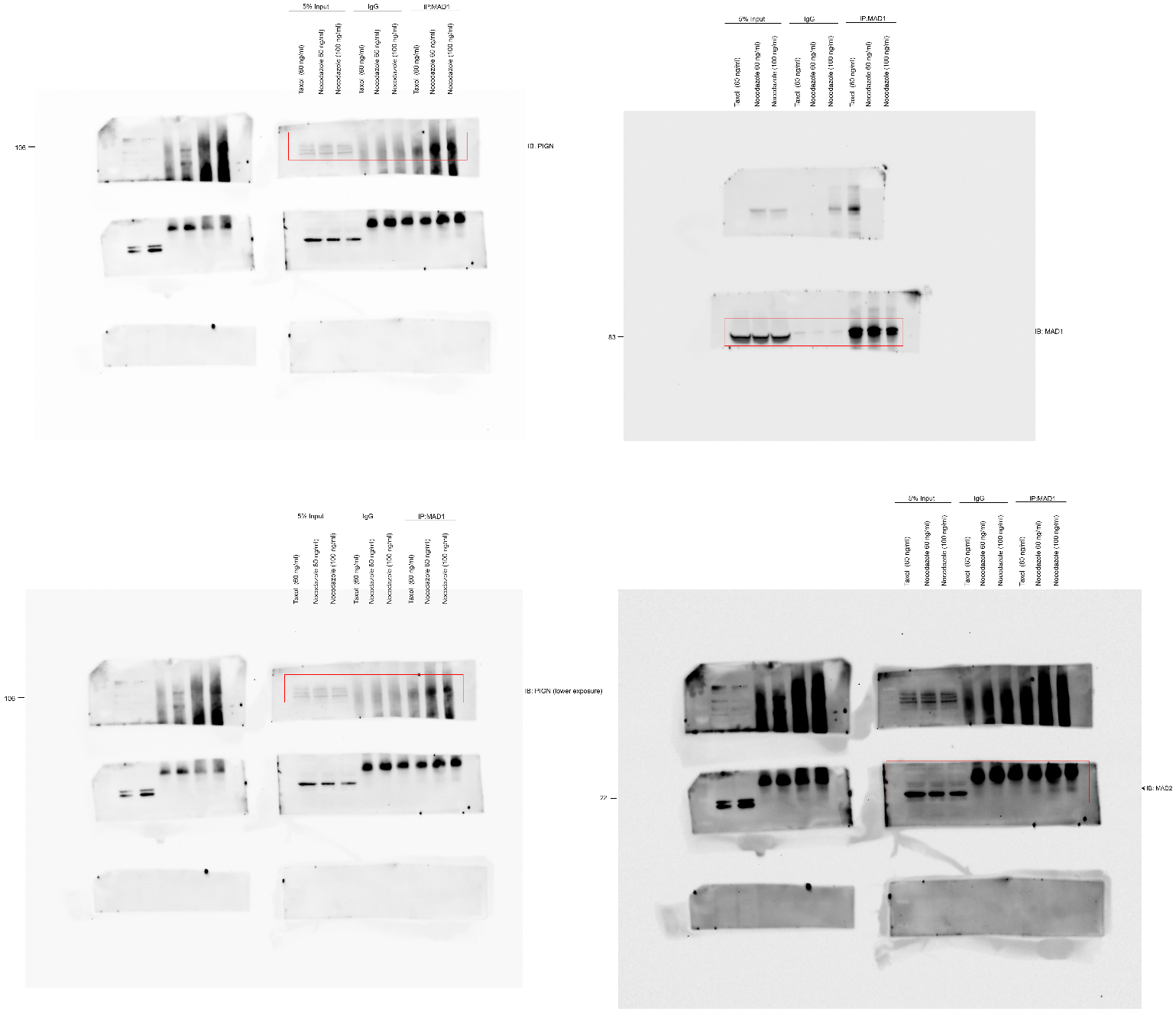


**Figure 3e**


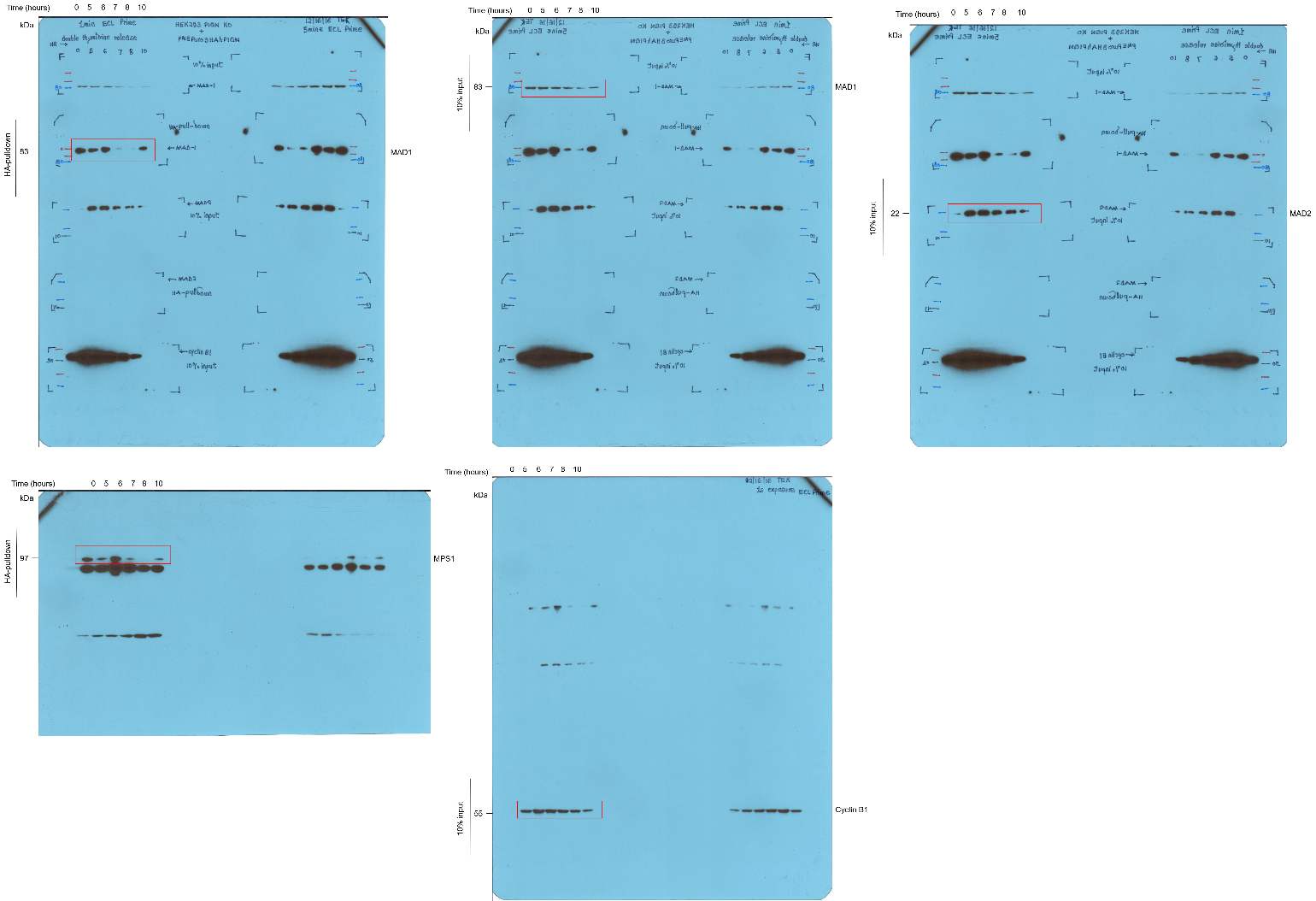


Full blot images are not presented for HA-PIGN (HA-pulldown and 10% input); representative blots shown in supplementary immunoblotting Figures 3a and b which were developed using the same antibody as indicated in the Western blot supplementary materials section.
